# Phosphorylation regulates the chromatin remodeler SMARCAD1 in nucleosome binding, ATP hydrolysis, and histone exchange

**DOI:** 10.1016/j.jbc.2024.107893

**Published:** 2024-10-17

**Authors:** Briana L. Aboulache, Nicole M. Hoitsma, Karolin Luger

**Affiliations:** 1 Department of Biochemistry, University of Colorado Boulder, Boulder, Colorado, USA; 2 Howard Hughes Medical Institute, Chevy Chase, Maryland, USA

**Keywords:** SMARCAD1, chromatin remodeling, phosphorylation, post-translational modification (PTM), nucleosome, histone chaperone, nucleosome assembly, ATP hydrolysis, histone exchange

## Abstract

Maintaining the dynamic structure of chromatin is critical for regulating the cellular processes that require access to the DNA template, such as DNA damage repair, transcription, and replication. Histone chaperones and ATP-dependent chromatin remodeling factors facilitate transitions in chromatin structure by assembling and positioning nucleosomes through a variety of enzymatic activities. SMARCAD1 is a unique chromatin remodeler that combines the ATP-dependent ability to exchange histones, with the chaperone-like activity of nucleosome deposition. We have shown previously that phosphorylated SMARCAD1 exhibits reduced binding to nucleosomes. However, it is unknown how phosphorylation affects SMARCAD1’s ability to perform its various enzymatic activities. Here we use mutational analysis, activity assays, and mass spectrometry, to probe SMARCAD1 regulation and to investigate the role of its flexible N-terminal region. We show that phosphorylation affects SMARCAD1 binding to nucleosomes, DNA, and histones H2A-H2B, as well as ATP hydrolysis and histone exchange. Conversely, we report only a marginal effect of phosphorylation for histone H3-H4 binding and nucleosome assembly. In addition, the SMARCAD1 N-terminal region is revealed to be critical for nucleosome assembly and histone exchange. Together, this work examines the intricacies of how phosphorylation governs SMARCAD1 activity and provides insight into its complex regulation and diverse activities.

The nucleosome is the fundamental repeating unit that packages genomic DNA into chromatin. It is composed of two copies each of the core histones H2A, H2B, H3, and H4, which form a histone octamer that is wrapped by ∼147 base pairs of DNA (1). This wrapping dramatically decreases DNA accessibility and creates both a barrier and a regulatory opportunity for the processes of DNA repair, transcription, and replication. The cell has evolved two classes of specialized protein complexes, histone chaperones and ATP-dependent chromatin remodeling factors (‘remodelers’), which facilitate dynamic access to packaged DNA and balance the competing requirements of DNA accessibility and compaction.

Under physiological conditions, the individual core histones are unstable and do not spontaneously form nucleosomes (2, 3). Histone chaperones bind histone heterodimers, shield their DNA binding surfaces to prevent aggregation and aberrant histone deposition, and ultimately facilitate nucleosome assembly (4, 5). Many histone chaperones assemble nucleosomes in a stepwise and ATP-independent manner, where the (H3-H4)_2_ tetramer is deposited onto the DNA first, followed by the addition of two H2A-H2B dimers (2, 4). The deposition of H2A-H2B dimers occurs either spontaneously or may depend on histone chaperones. After their deposition, the position and composition of nucleosomes can be adjusted by ATP-dependent chromatin remodelers. These enzymes make up a large and diverse class which use ATP hydrolysis to disrupt DNA-histone interactions, thereby altering chromatin structure by translocating, ejecting, or exchanging histones (6, 7, 8). Chromatin remodelers are grouped into four different subfamilies based on their subunits and catalytic ATPase domains. Although the ATPase domain is highly conserved across all remodeler families, members in each family contain other unique domains and subunits that are responsible for determining various remodeling activities.

SMARCAD1 is a member of the INO80 chromatin remodeler family that combines the ATP-dependent ability to exchange histones, with the chaperone-like, ATP-independent activity to deposit histones and assemble nucleosomes (9). The INO80 remodeler family (including SMARCAD1) is characterized by a “split” ATPase domain that contains a long insertion between the two ATPase lobes (7). In addition, SMARCAD1 contains two CUE domains that are thought to be responsible for protein interactions and recruitment to sites of DNA damage (10, 11, 12). While most members of the INO80 subfamily function as multi-subunit complexes (up to 590 kDa), which contributes to their regulation, SMARCAD1 consists of only a single 120 kDa subunit (13). The ATPase domain of human SMARCAD1 is highly conserved across different species including mouse, *Xenopus*, *Drosophila*, yeast, and *C. elegans* (Fig. S1). SMARCAD1 and its orthologs function in several cellular contexts, having been reported to both evict nucleosomes to form euchromatin and to deposit nucleosomes to maintain heterochromatin. While classified as a chromatin remodeler, SMARCAD1 is unusual as it possesses both ATP-dependent remodeler activity to break DNA-histone contacts and ATP-independent chaperone-like activity to assemble *de novo* nucleosomes (9). As such, SMARCAD1, like other remodeling factors, likely requires precise regulation (14, 15). Mutation and knockdown in cells leads to genomic instability and is associated with breast cancer and autosomal-dominant inherited diseases, further highlighting its biological significance (15, 16, 17, 18, 19).

We showed previously that SMARCAD1 is phosphorylated at over 20 residues (*i.e.* phosphosites) *in vitro*, all of which have been confirmed *via* cellular phosphoproteomic studies of human SMARCAD1 (20, 21, 22, 23, 24, 25, 26, 27, 28, 29, 30, 31, 32, 33, 34, 35, 36, 37, 38, 39, 40, 41, 42, 43, 44, 45, 46, 47, 48, 49, 50, 51, 52, 53, 54, 55, 56, 57, 58, 59, 60, 61, 62, 63, 64, 65). Phosphorylated human SMARCAD1 (purified from insect cells) shows reduced binding to nucleosomes *in vitro* (9). However, upon phosphatase treatment, dephosphorylated SMARCAD1 binds to nucleosomes and uses the energy from ATP hydrolysis to extract histones from a nucleosome and deposit them onto a new DNA fragment (9). In addition to this *in vitro* study, numerous *in vivo* studies report the importance of human SMARCAD1 phosphorylation, by affecting its localization and interactions. Phosphorylation at specific sites (T71 and T906) was suggested to control its recruitment to DNA damage and its interactions with specific repair proteins (12, 60, 66, 67). Both human SMARCAD1 and Fun30, the yeast homolog, have been identified as targets of cyclin-dependent kinase (CDK), which may provide a means of cell cycle regulation (66, 68). Additionally, SMARCAD1 has been shown to be phosphorylated by ataxia–telangiectasia mutated (ATM) kinase upon DNA damage (12, 60, 67). These studies have identified specific phosphosites in human SMARCAD1, but how global phosphorylation affects SMARCAD1 chromatin remodeling and its various functions is unknown (12, 60, 66, 67, 68).

Remodelers in other subfamilies have also been shown to be regulated by phosphorylation (69). Human SWI/SNF, isolated at various stages of the cell cycle is phosphorylated and inactive, with a restoration of remodeling activity upon dephosphorylation (69). In addition to post-translational modifications, many remodelers are regulated through autoinhibition to prevent rampant remodeling activities. The SMARCAD1 yeast homolog, Fun30, has an evolutionarily conserved domain on the N-terminal region containing a SAM-like fold (termed SAM-key) that is important for its function in DNA repair and gene silencing (70). This *in vitro* study revealed that removing the SAM-key (analogous to amino acids 383–497 of human SMARCAD1) resulted in a loss of Fun30 ATP hydrolysis, histone sliding, and histone eviction (70). This work suggests that the SAM-key acts as a regulatory element that controls ATP hydrolysis and motor activity, similar to Protrusion I in several other remodelers (RSC, SWI/SNF, Snf2, and ISWI) (70, 71, 72, 73). Together, these studies suggest a conserved allosteric mechanism controlling ATP hydrolysis.

Here, we use mutational analysis, activity assays, and mass spectrometry to probe SMARCAD1 regulation and to investigate the role of its flexible N-terminal region. We identified phosphorylation as a regulatory mechanism for SMARCAD1 binding to nucleosomes, DNA, and histone H2A-H2B, as well as for ATP hydrolysis and histone exchange from undersaturated nucleosomes. Conversely, we report only a marginal effect of phosphorylation for histone H3-H4 binding and nucleosome assembly. In addition, the SMARCAD1 N-terminal region is revealed to be critical for nucleosome assembly and histone exchange. By further characterizing the mechanism and regulation of SMARCAD1, we can begin to understand how post-translation modifications (PTMs) influence chromatin remodeler interaction with nucleosomes and enzymatic activity, ultimately modulating gene expression and silencing.

## Results

### Eliminating known phosphosites only partially restores the ability of SMARCAD1 to bind to nucleosomes

At present, there are 23 known phosphorylation sites on SMARCAD1 and we have shown that SMARCAD1 phosphorylation state affects its ability to bind to nucleosomes (9). Here, we further analyze the effect of phosphorylation by comparing the activities of the phosphorylated (P) and phosphatase (CIP)-treated dephosphorylated (deP) forms of SMARCAD1 (Fig. S2). We hypothesized that by mutating the 23 known phosphosites, we could generate a constitutively active SMARCAD1 protein that could not be phosphorylated (and deactivated). To this end, we made a version of SMARCAD1 where each of the 23 phosphorylation sites was mutated to alanine (23A SMARCAD1) (Fig. S2).

**Figure 1:**
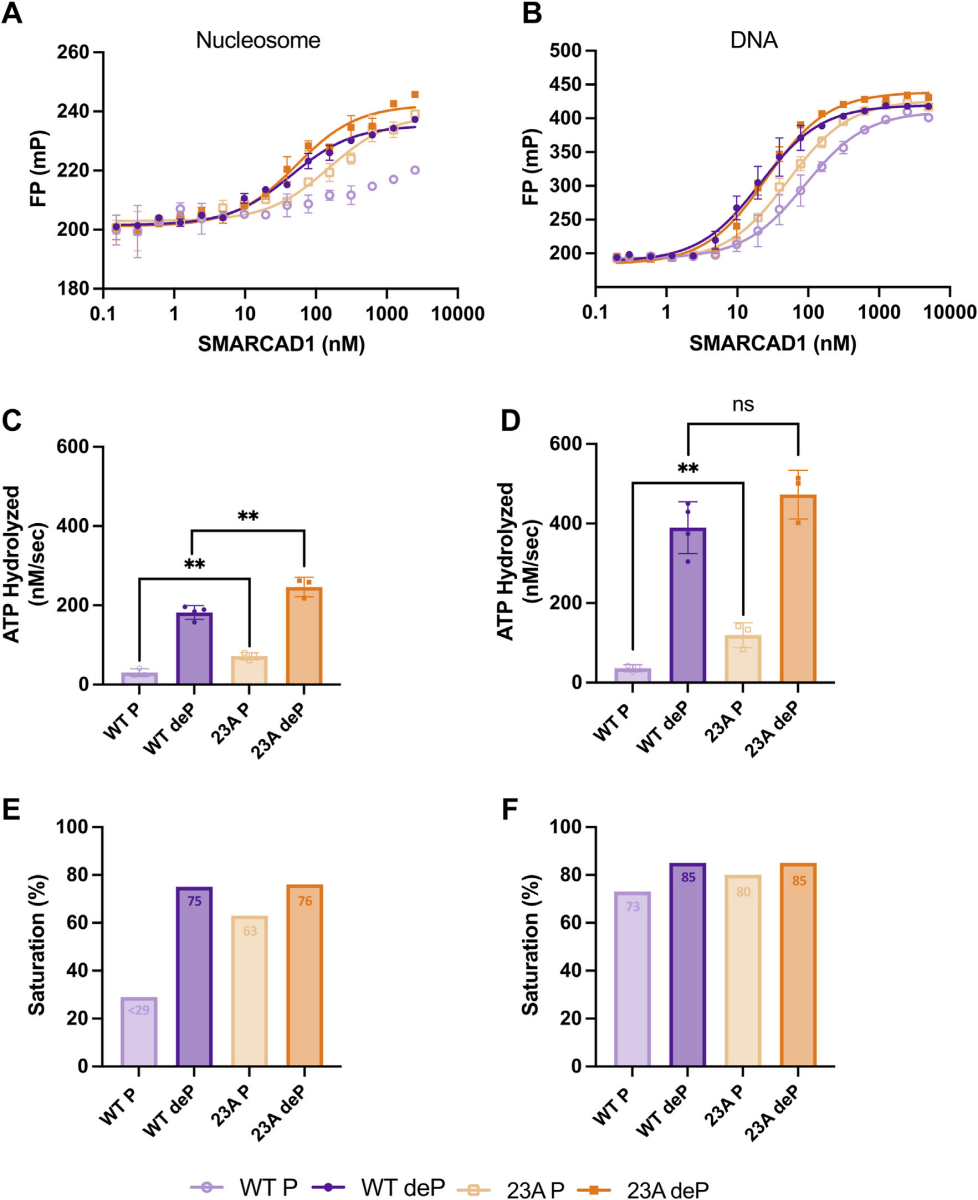
**Binding analysis and ATP hydrolysis activity of WT and 23A SMARCAD1.***A* and *B*, fluorescence polarization (FP) assay of WT and 23A SMARCAD1 in phosphorylated (P) and dephosphorylated (deP) forms with (*A*) Alexa Fluor 488–labeled nucleosome (10 nM) or (*B*) Alexa Fluor 488/647–labeled 165 DNA (10 nM). Data shown are one representative curve (error bars represent technical duplicate). Curve fits shown have R^2^ ≥ 0.89. Data are summarized in Table 1. *C* and *D*, the rate of WT and 23A SMARCAD1 ATP hydrolysis in the presence of (*C*) nucleosome or (*D*) DNA. All are the mean ± SD from ≥ three replicates (shown as individual points). ∗∗*p* ≤ 0.01 and nonsignificant (ns); unpaired *t* test. Data are summarized in Table 2. Refer to Fig. S6 for representative raw data. *E* and *F*, percent saturation under ATPase assay conditions in the presence of (*E*) nucleosome or (*F*) DNA.

We first tested wild type (WT) and 23A SMARCAD1 in their phosphorylated and dephosphorylated forms for their ability to interact with nucleosomes. For this assay, histone octamer with an Alexa-488 fluorophore on histone H4E63C was assembled onto a 165-base pair (bp) DNA to form a nucleosome (601 positioning sequence flanked by 7 and 11 bp of DNA on either end). To quantify SMARCAD1 binding, fluorescence polarization (FP) of the labeled nucleosome was monitored at increasing concentrations of SMARCAD1 (Figs. 1*A* and S3). Consistent with our previous publication (9), WT SMARCAD1 was only able to bind nucleosomes in its dephosphorylated form (with an affinity of 85 nM), while reduced binding was observed for phosphorylated SMARCAD1 (no fit; Fig. 1*A* and Table 1). In contrast, lower affinity binding is observed for 23A SMARCAD1 in the phosphorylated form (213 nM), while phosphatase treatment restores binding to wild type levels (71 nM) (Fig. 1*A* and Table 1). Importantly, only a partial recovery of binding by the phosphorylated 23A mutant suggests that this mutant is still partially inhibited even when the 23 known phosphosites are unavailable for modification. Additionally, these data demonstrate that the 23 mutated amino acids in the N-terminal region do not appreciably contribute to nucleosome binding.

**Table 1:** SMARCAD1 binding constants

SMARCAD1	Phosphorylation state	165 Nucs (nM)	DNA (nM)	H2A-H2B (nM)	H3-H4 (nM)
WT	P	No Fit	98 ± 8	23 ± 8	151 ± 78
	deP	85 ± 38	26 ± 9	131 ± 72	171 ± 63
23A	P	213 ± 36	49 ± 12	100 ± 42	160 ± 93
	deP	71 ± 7	28 ± 2	222 ± 20	122 ± 14
Δ350	P	No Fit	105 ± 12	150 ± 67	495 ± 114
	deP	114 ± 27	60 ± 18	No Fit	No Fit

Binding constants for WT SMARCAD1, 23A SMARCAD1, and Δ350 SMARCAD1 in their respective phosphorylated and dephosphorylated forms. Data is mean ± SD from ≥ three replicates and fit of R^2^ ≥ 0.89.

To test how phosphorylation alters the ability of WT and 23A SMARCAD1 to interact with the nucleosomal components (*i.e.* DNA and histones), we used a fluorescently labeled 165-bp DNA, labeled H2B T115C-H2A histone complex, or labeled H4 E63C-H3 histone complex to monitor DNA or histone binding *via* FP. As was observed for nucleosome binding, WT SMARCAD1 binds DNA with a higher affinity in its dephosphorylated form (26 nM) compared to the phosphorylated form (98 nM), and 23A SMARCAD1 binding to DNA depends to a lesser extent on dephosphorylation (28 vs 49 nM; Figs. 1*B*, S4, and Table 1). Both WT and 23A SMARCAD1 bind to H2A-H2B histones; however, phosphorylation increases binding by 6-fold and 2-fold, respectively, compared to the dephosphorylated states (Fig. S5 and Table 1). This is likely due to the additional negative charges from the phosphate groups which could favorably interact with the positively charged H2A-H2B histones. A similarly moderate effect of phosphorylation is observed for 23A SMARCAD1 (Fig. S5 and Table 1). This further suggests that the removal of some residues that can harbor negatively charged phosphate groups results in a weaker binding affinity. In contrast, WT and 23A SMARCAD1 both bind H3-H4 histones with similar affinity in their phosphorylated and dephosphorylated form (Fig. S5 and Table 1), indicating the histone H3-H4 interaction is not affected by the phosphorylation state of SMARCAD1. As observed for H2A-H2B binding, the 23 amino acids that were mutated do not contribute to the interaction with H3-H4.

These experiments confirm that SMARCAD1 affinity for nucleosomes is greatly diminished by phosphorylation, but that phosphorylation has relatively minor effects on DNA and histone H2A-H2B binding, and no effect on histone H3-H4 binding. Importantly, mutating the 23 known phosphorylation sites in SMARCAD1 reduces but does not completely abolish the dependence on phosphatase treatment for nucleosome and DNA binding.

### Phosphorylation regulates SMARCAD1 ATPase activity

SMARCAD1 ATP hydrolysis is activated by either nucleosomes or DNA (9). To measure ATPase activity, we used an assay that measures NADH oxidation (change in A340) as a readout of ATP hydrolysis upon addition of coupling enzymes, pyruvate kinase and lactate dehydrogenase. Under these conditions, phosphorylated WT SMARCAD1 hydrolyzes very little ATP in the presence of either nucleosomes or DNA. Upon dephosphorylation, SMARCAD1 displays robust ATPase activity in the presence of either activator (Figs. 1, *C* and *D*, S6, and Table 2). To distinguish between changes in ATPase activity attributed to either catalytic defects or reduced binding of activators required for activity, we calculated the percent saturation of SMARCAD1 under the ATPase assay conditions using the experimentally determined Kd values (Fig. 1, *E* and *F*). This analysis reveals that for phosphorylated SMARCAD1 with nucleosomes, only a small fraction of SMARCAD1 is bound (<29%) and its lack of ATPase activity is most likely due to a defect in binding the nucleosome activator rather than a catalytic defect (Fig. 1*E*). In contrast, both phosphorylated and dephosphorylated WT SMARCAD1 are mostly DNA-bound, with 73% and 85% saturation, respectively (Fig. 1*F*). Nevertheless, the phosphorylated form lacks ATPase activity. This suggests that while phosphorylation does not affect DNA binding in WT SMARCAD1, it does inhibit ATP hydrolysis, indicating a catalytic defect when phosphorylated. We previously reported that with excess activator (100 nM SMARCAD1 to 1 *μ*M DNA or nucleosome), nucleosomes are more potent than DNA in activating SMARCAD1 (9). Here, at an equimolar ratio (1 *μ*M SMARCAD1 to 1 *μ*M DNA or nucleosome), which yields better signal-to-noise ratios, we observe only a 2-fold difference in ATP hydrolysis between activators.

**Table 2:** SMARCAD1 enzymatic activity

SMARCAD1	Phosphorylation state	ATP hydrolysis		*De novo* nucleosome assembly		Histone exchange	
		Nuc (nM/s)	DNA (nM/s)	K_assembly_ (μM^-1^)	Maximum product (RFU)	k_exchange_ (sec^-1^ × 10^-3^)	Maximum product (RFU)
WT	P	30.0 ± 9.7	36.2 ± 8.9	0.7 ± 0.5	0.9 ± 0.1	1.1 ± 0.3	0.1 ± 0.03
	deP	182.0 ± 17.1	389.7 ± 65.3	1.3 ± 0.6	0.9 ± 0.03	2.3 ± 0.3	0.9 ± 0.1
23A	P	71.6 ± 9.4	119.5 ± 31.4	0.8 ± 0.02	0.9 ± 0.2	0.6 ± 0.4	0.4 ± 0.03
	deP	246.2 ± 24.3	472.5 ± 61.3	1.1 ± 0.4	0.8 ± 0.2	1.8 ± 0.2	0.9 ± 0.1
Δ350	P	118.4 ± 13.5	37.2 ± 1.3	1.1 ± 0.7	0.6 ± 0.1	1.6 ± 0.2	0.4 ± 0.2^‡^
	deP	151.7 ± 15.5	78.3 ± 15.3	1.1 ± 0.6	0.6 ± 0.1	1.6 ± 0.3	0.1 ± 0.01

Kinetic parameters for WT SMARCAD1, 23A SMARCAD1, and Δ350 SMARCAD1 in their respective phosphorylated and dephosphorylated forms. Phosphorylated Δ350 SMARCAD1 produces a diffuse nucleosome-like product of unknown composition (‡) in histone exchange. All parameters are the mean ± SD from ≥ three replicates and fit of R^2^ ≥ 0.89.

A larger fraction of phosphorylated 23A SMARCAD1 binds to nucleosomes (63%) compared to WT SMARCAD1, likely contributing to a partial restoration of ATPase activity (Fig. 1*E*). Similarly to WT SMARCAD1, phosphorylated and dephosphorylated 23A SMARCAD1 binds to DNA with 80% and 85% saturation, respectively (Fig. 1*F*). Because phosphorylated 23A SMARCAD1 binds to DNA but lacks robust ATPase activity, this suggests that there is a catalytic defect when phosphorylated that is partially restored upon mutation of the phosphorylated sites. However, dephosphorylation of the 23A mutant further increased ATP hydrolysis with nucleosome and DNA to the level of dephosphorylated wild type SMARCAD1 (Fig. 1, *C* and *D*, and Table 2). This further supports the idea that ATPase activity is regulated by additional phosphosites beyond the 23 mutated in 23A SMARCAD1.

### Removing known phosphosites only partially restores SMARCAD1 exchange activity and does not alter SMARCAD1-dependent nucleosome assembly

**Figure 2:**
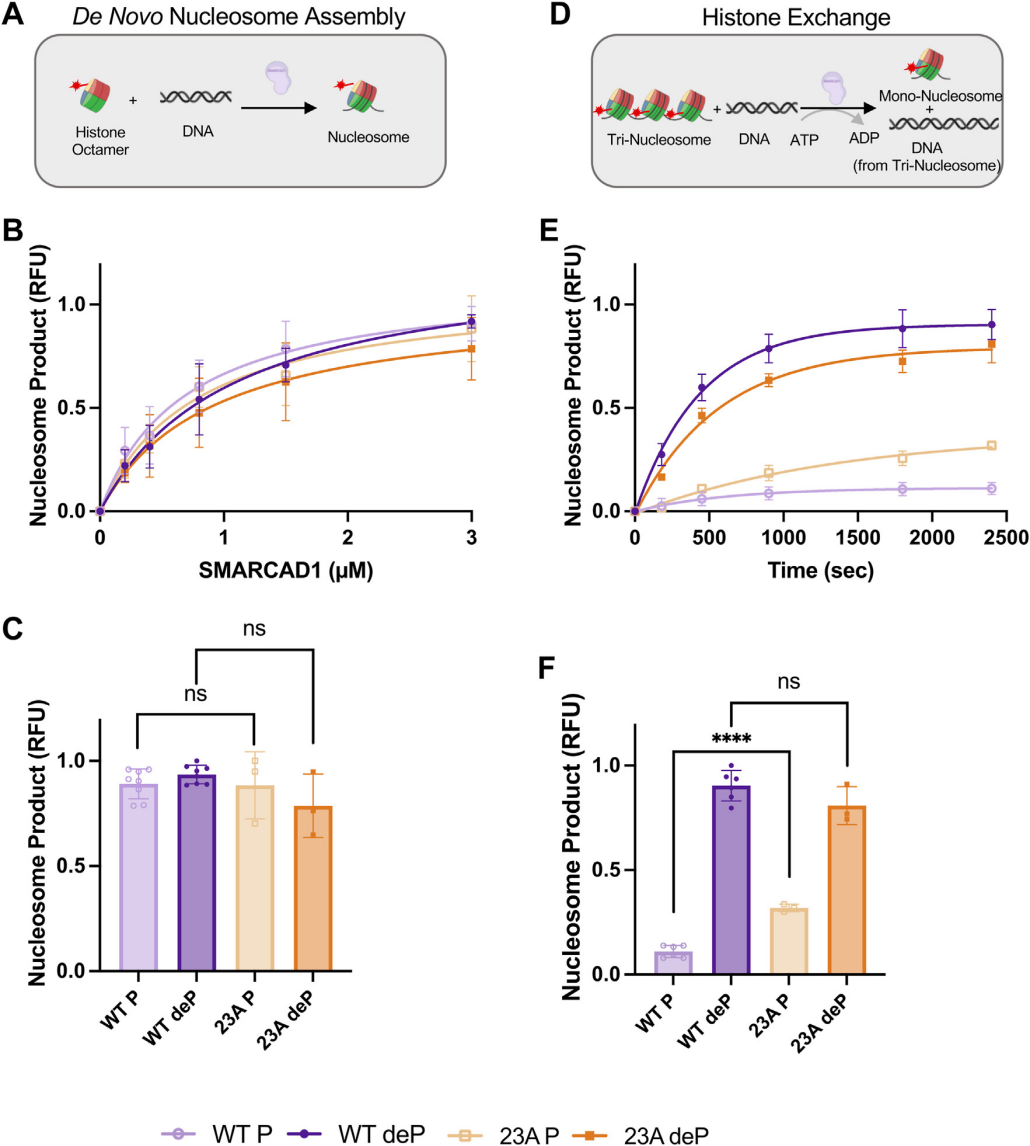
***De novo* nucleosome assembly and histone exchange activity of WT and 23A SMARCAD1.***A*, schematic for SMARCAD1 *de novo* nucleosome assembly assay. SMARCAD1 was mixed with labeled octamer and DNA. Samples were run on a native gel and the formation of labeled mono-nucleosome was monitored. *B*, quantification with fits for *de novo* nucleosome assembly experiments with (*C*) maximum amount of nucleosome formation (upon the addition of 3 *μ*M SMARCAD1), quantified as relative fluorescence unit (RFU, product formation relative to dephosphorylated WT SMARCAD1) determined from experiments as shown in Fig. S7. *D*, schematic for SMARCAD1 histone exchange assay. SMARCAD1 was mixed with labeled tri-nucleosome and DNA. Reactions were initiated with ATP and quenched with EDTA at varying timepoints. The samples were run on a native gel and the formation of labeled mono-nucleosome was monitored. *E*, quantification with fits for histone exchange experiments with (*F*) maximum nucleosome product formation, quantified as relative fluorescence unit (RFU, product formation relative to dephosphorylated WT SMARCAD1) determined from experiments as shown in Fig. S9. All are the mean ± SD from ≥ three replicates (shown as individual points). Curve fits shown have R^2^ ≥ 0.89. ∗∗∗∗*p* ≤ 0.0001 and nonsignificant (ns); unpaired *t* test. Refer to Fig. S9 for representative raw data. Data for all panels are summarized in Table 2.

We used our established assays to determine the effect of phosphorylation on SMARCAD1 nucleosome assembly and histone exchange activities (9). For nucleosome assembly, SMARCAD1 deposits histones onto DNA to form nucleosomes in an ATP-independent manner. In this assay, we incubated SMARCAD1 with a histone octamer containing a fluorescent label on H2B, then added a 147 bp DNA fragment (Fig. 2*A*). The formation of fluorescent mono-nucleosomes was quantified and normalized to the maximum product formation from WT dephosphorylated SMARCAD1 (Figs. 2*B*, S7, and Table 2). Fitting these data as a function of SMARCAD1 concentration reveals the amount of SMARCAD1 required to assemble 50% nucleosome product relative to the maximum nucleosome formation (listed as K_assembly_ in Table 2). As an additional parameter, we also compared the maximum amount of nucleosome formation (at 3 *μ*M SMARCAD1) (Fig. 2*C* and Table 2). This analysis shows that *de novo* nucleosome assembly is not affected by SMARCAD1 phosphorylation state: it takes the same amount of WT SMARCAD1 to assemble 50% nucleosome product (K_assembly_), and similar product yield, with a less than 2-fold difference from phosphatase treated SMARCAD1 (Fig. 2, *B* and *C*, and Table 2). Furthermore, both phosphorylated and dephosphorylated 23A SMARCAD1 assemble nucleosomes with a similar K_assembly_ and product yield as WT SMARCAD1 (<2-fold difference) (Fig. 2, *B* and *C*, and Table 2). This aligns with our finding that histone binding is not hindered by either phosphorylation or mutation of the 23 known phosphorylation sites.

We previously showed that SMARCAD1 removes histones from a nucleosome in an ATP-dependent manner and re-assembles them onto a different DNA fragment (*i.e.* histone exchange) (9). In this histone exchange assay, we utilize a tri-nucleosome with a fluorescent label on H2B to monitor ATP-dependent formation of mono-nucleosome on a 147 bp DNA acceptor (Fig. 2*D*). For assay optimization, we tested SMARCAD1 histone exchange activity on multiple tri-nucleosome substrates. Further analysis of the substrates *via* mass photometry revealed variation in tri-nucleosome saturation that correlates with SMARCAD1 histone exchange activity (Fig. S8). This data reveals that SMARCAD1 is better at ATP-dependent histone exchange when provided with an undersaturated tri-nucleosome substrate. Furthermore, when the tri-nucleosome substrate is oversaturated (*i.e.* histones exceeding three histone octamers are bound to the assemblies) we observe an ATP-independent nucleosome assembly activity in which SMARCAD1 is able to remove these histones and assemble nucleosomes in the absence of ATP (Fig. S8). Thus, for the histone exchange assay, we utilized an undersaturated tri-nucleosome substrate for maximum ATP-dependent SMARCAD1 histone exchange activity. Reactions were quenched with EDTA at the indicated time points, and the formation of fluorescent mono-nucleosomes was quantified and normalized to the maximum product formation from dephosphorylated WT SMARCAD1 (Fig. S9). The time course of nucleosome product formation was fit to determine the rate of histone exchange (k_exchange_) (Fig. 2*E* and Table 2). The amount of nucleosome product at the final timepoint was also used to compare maximum nucleosome formation (Fig. 2*F* and Table 2). In its phosphorylated form, WT SMARCAD1 produces 10-fold less nucleosome product than in the dephosphorylated state. This was expected based on its inability to bind nucleosomes and hydrolyze ATP, both required for histone removal. As previously published (9), a robust exchange activity was observed for dephosphorylated WT SMARCAD1 (Fig. 2, *E* and *F*, and Table 2). Phosphorylated 23A SMARCAD1 has higher histone exchange activity than phosphorylated wild type, again indicating a partial recovery of activity, which can be fully restored upon phosphatase treatment (Fig. 2, *E* and *F*, and Table 2).

### Mass spectrometry reveals additional phosphorylation sites on SMARCAD1

**Figure 3:**
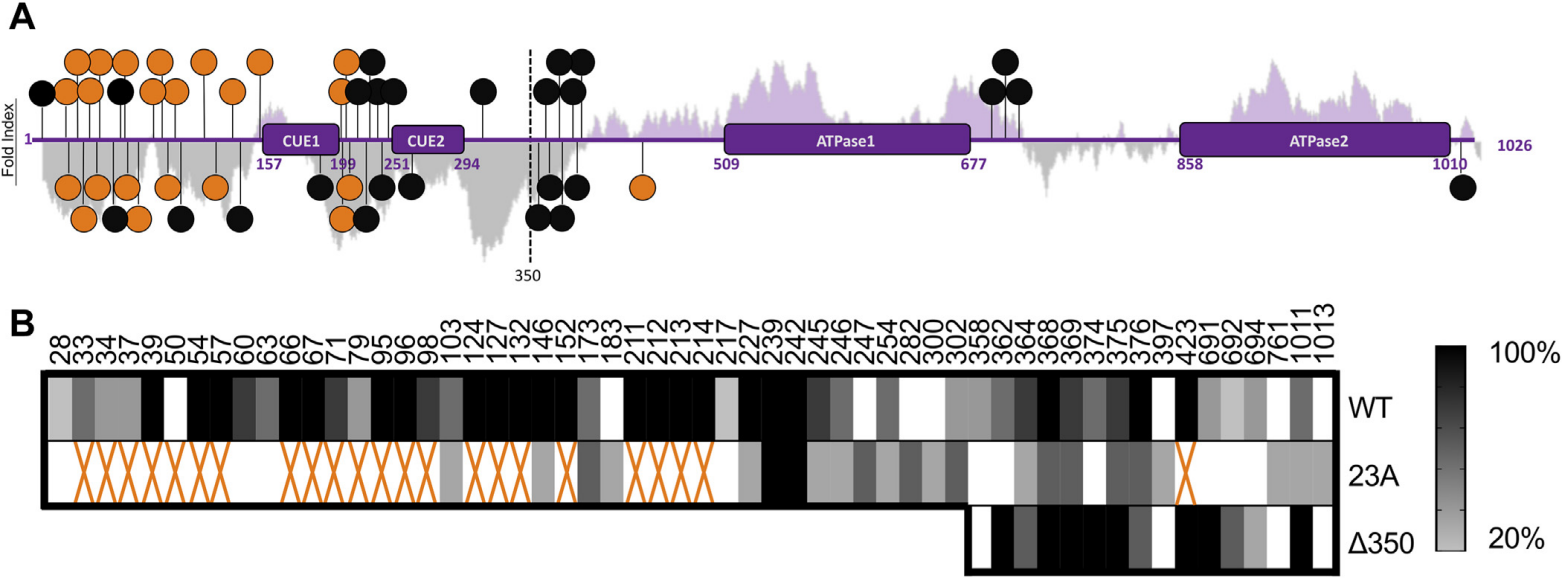
**Mass spectrometry reveals additional phosphorylation sites on SMARCAD1.***A*, schematic of known SMARCAD1 domains. Predicted protein-folding states using FoldIndex indicate regions of disorder (*gray*) and order (*purple*) with the amplitude indicating the degree of order/disorder. WT SMARCAD1 phosphosites are represented as *circles*, with previously reported phosphosites shown in *orange* (reported in ref 9) and additional phosphosites identified in this study in *black*. *B*, visualization of phosphosites identified *via* mass spectrometry analysis of WT SMARCAD1 (n = 5), 23A SMARCAD1 (n = 3), and Δ350 SMARCAD1 (n = 3). The darker the color represents that a site was found in a higher percent of biological replicates. Previously reported phosphosites (that were mutated to alanine in the 23A mutant) are annotated with an *orange* X.

To probe for any additional phosphorylation sites on SMARCAD1, we analyzed trypsin-digested SMARCAD1 on an Orbitrap Q-Exactive HF-X instrument (74). The increased sensitivity of this instrument compared to LTQ-Orbitrap Velos allowed the identification of 26 previously unidentified phosphorylation sites in WT SMARCAD1 (Fig. 3*A*). Out of the 163 residues that can potentially be phosphorylated on SMARCAD1 (serine, threonine, and tyrosine residues), we have observed 49 to be phosphorylated in SMARCAD1 prepared from insect cell culture (Fig. 3*A*). Of these, 36 are distributed throughout the N-terminal region and 13 are within the C-terminal region harboring the ATPase domains. Variations between multiple biological replicates suggests heterogeneity in phosphorylation as not all sites were found in every replicate (Fig. 3*B*), likely due to stochastic differences in SMARCAD1 expression and preparation. Variability in peak identification during mass spectrometry data collection may also contribute to the apparent heterogeneity in phosphorylation sites.

We also analyzed 23A SMARCAD1 *via* trypsin-digested mass spectrometry to directly probe for the hypothesized additional phosphosites. This analysis revealed 23 phosphorylation sites remaining on the 23A mutant. Interestingly, seven of these were not observed for WT SMARCAD1 (Fig. 3*B*). These additional sites provide an explanation as to why 23A SMARCAD1 activity is still partially inhibited without phosphatase treatment.

### The N-terminal region of SMARCAD1 is required to assemble *de novo* nucleosomes and exchange histones

Predicted protein folding using FoldIndex and AlphaFold3 indicate regions of unstructured and structured regions within SMARCAD1 (Figs. 3*A* and S10). These analyses show the N-terminal region (∼350 residues) to be predominantly disordered, apart from the two structured CUE domains. The C-terminal portion of SMARCAD1 (amino acid 351–1026) contains the structured split ATPase domain, as anticipated from homology modeling. Moreover, the N-terminal region was not visible in cryoEM maps of the SMARCAD1-nucleosome complex, providing further evidence that it is disordered and likely does not engage the nucleosome substrate (9). The individual ATPase domain lobes 1 and 2 represented in this low-resolution structure superimpose with the Alphafold3 model with RMSD values of 4.2 and 4.3 Å, respectively.

To probe the contribution of the flexible N-terminal region to the various SMARCAD1 interactions and activities, we prepared a SMARCAD1 mutant where the N-terminal 350 amino acids were deleted (Δ350 SMARCAD1) (Fig. S2). Mass spectrometry of trypsin-digested Δ350 SMARCAD1 reveals 12 remaining phosphosites, consistent with the C-terminal sites found on WT SMARCAD1 (Fig. 3*B*). This truncated version of SMARCAD1 (at 78 kDa) is within the range for native MS, which revealed the presence of 3 to 11 phosphates (Fig. S11). Although this is consistent with our digested MS data, the observed spectrum of phosphorylation states highlights the phosphorylation heterogeneity of the SMARCAD1 sample also observed *via* digested MS (Fig. 3*B*).

**Figure 4:**
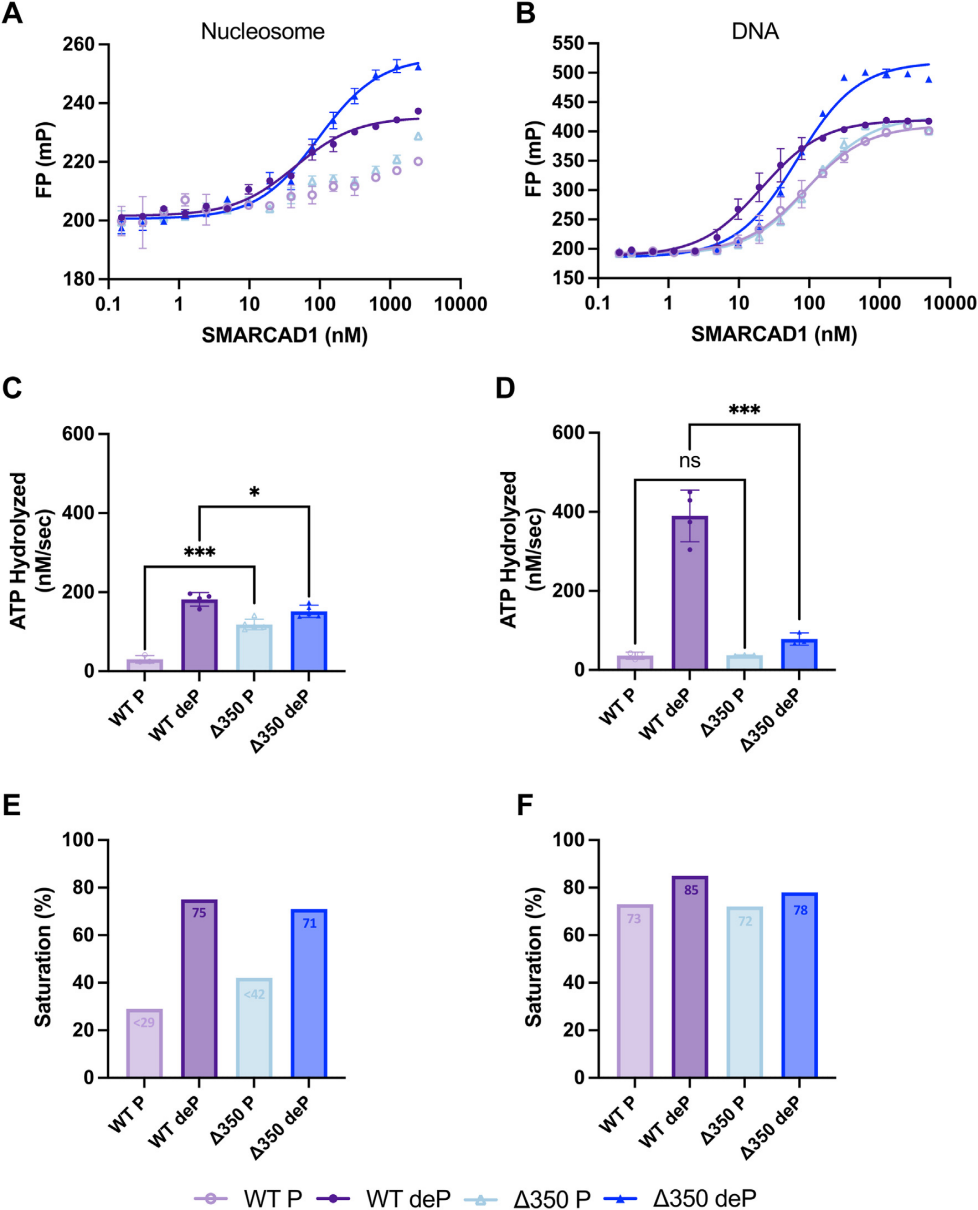
**Binding analysis and ATP hydrolysis activity of WT and Δ350 SMARCAD1.***A* and *B*, fluorescence polarization (FP) assay of WT and Δ350 SMARCAD1 in phosphorylated (P) and dephosphorylated (deP) forms with (*A*) Alexa Fluor 488–labeled nucleosome (10 nM) or (*B*) Alexa Fluor 488/647–labeled 165 DNA (10 nM). Data shown are one representative curve (error bars represent technical duplicate). Curve fits shown have R^2^ ≥ 0.89. Data are summarized in Table 1. *C* and *D*, the rate of WT and Δ350 SMARCAD1 ATP hydrolysis in the presence of (*C*) nucleosome or (*D*) DNA. All are the mean ± SD from ≥ three replicates (shown as individual points). ∗∗∗*p* ≤ 0.001, ∗*p* ≤ 0.05 and nonsignificant (ns); unpaired *t* test. Data are summarized in Table 2. Refer to Fig. S6 for representative raw data. *E* and *F*, percent saturation under ATPase assay conditions in the ATPase assay in the presence of (*E*) nucleosome or (*F*) DNA.

To test if the N-terminal region is required for SMARCAD1 binding, we assayed the ability of the Δ350 mutant to bind nucleosomes, DNA, and histones, in both its phosphorylated and dephosphorylated state. For this mutant, nucleosome binding was only observed in the dephosphorylated state (114 nM, Figs. 4*A*, S3, and Table 1). This is similar to what was observed for WT SMARCAD1, suggesting that the C-terminal phosphorylation sites are responsible for inhibition of nucleosome binding, and demonstrating that the 350 N-terminal amino acids do not contribute to nucleosome binding. Phosphorylated Δ350 SMARCAD1 binds to DNA with similar affinity as phosphorylated WT SMARCAD1, but only a modest increase in affinity is observed upon dephosphorylation (Figs. 4*B*, S4, and Table 1). Intriguingly, Δ350 SMARCAD1 can only interact with histones H2A-H2B (150 nM) and H3-H4 (495 nM) in its phosphorylated form (Fig. S5 and Table 1). Together, these data show that the N-terminal region is not involved in the interaction with nucleosomes, but its removal partially decreases DNA binding and greatly decreases histone binding. Our data also suggest the presence of regulatory phosphosites in the structured C-terminal region of SMARCAD1 promote histone binding but inhibit nucleosome binding. The difference in the requirement for dephosphorylation for interactions with nucleosome, DNA, and histones also suggests that different regions of SMARCAD1 are implicated in nucleosome binding *versus* binding to histone complexes or DNA.

To determine the importance of the N-terminal domain for SMARCAD1 enzymatic functions, we analyzed the activity of the Δ350 mutant in each of the SMARCAD1 assays described above. ATP hydrolysis of both forms of Δ350 SMARCAD1 can be activated by nucleosomes to near dephosphorylated WT levels, suggesting that the deletion of the N-terminal domain eliminates the requirement for dephosphorylation (Fig. 4*C* and Table 2). As observed for WT SMARCAD1, this activity is likely effected by reduced nucleosome binding in the phosphorylated state (Fig. 4*E*). Remarkably, neither form of Δ350 SMARCAD1 is robustly activated by DNA to perform ATP hydrolysis, despite the ability to bind DNA (Fig. 4, *D* and *F*, and Table 2). This suggests that removing the N-terminal region results in a catalytic defect upon addition of DNA activator.

**Figure 5:**
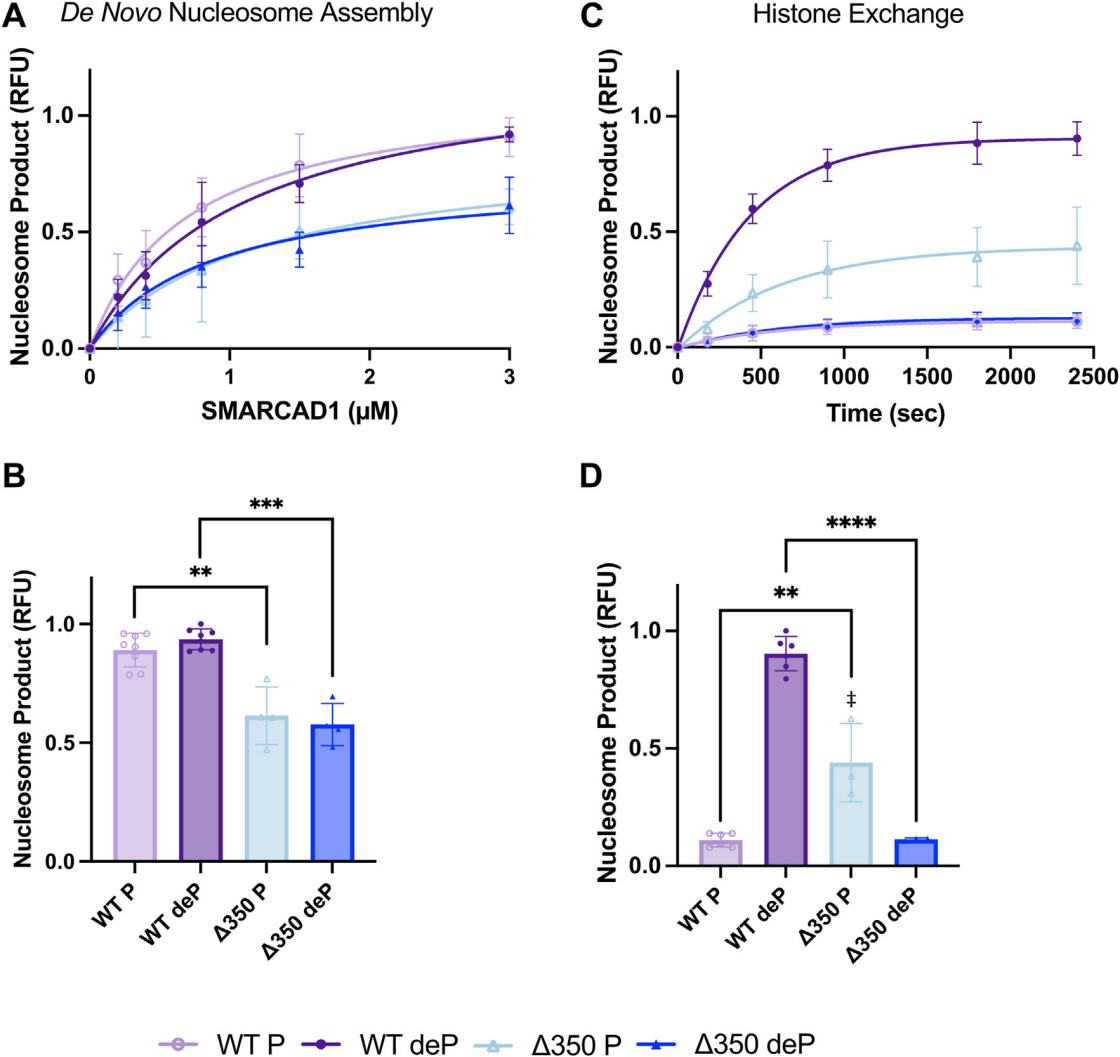
***De novo* nucleosome assembly and histone exchange activity of WT and Δ350 SMARCAD1.***A*, quantification with fits for nucleosome assembly experiments with (*B*) maximum amount of nucleosome formation (upon the addition of 3 *μ*M SMARCAD1), quantified as relative fluorescence unit (RFU, product formation relative to dephosphorylated WT SMARCAD1) determined from experiments as shown in Fig. S7. *C*, quantification with fits for histone exchange experiments with (*D*) maximum nucleosome product formation, quantified as relative fluorescence unit (RFU, product formation relative to dephosphorylated WT SMARCAD1). Δ350 SMARCAD1 produces a diffuse nucleosome-like product of unknown composition (‡). All are the mean ± SD from ≥ three replicates (shown as individual points). Curve fits shown have R^2^ ≥ 0.89. All WT SMARCAD1 is the data represented in Fig. 2, shown here for comparison to Δ350 SMARCAD1 mutant. ∗∗∗∗*p* ≤ 0.0001, ∗∗∗*p* ≤ 0.001, and ∗∗*p* ≤ 0.01; unpaired *t* test. Refer to Fig. S9 for representative raw data. Data for all panels are summarized in Table 2.

For *de novo* nucleosome assembly, the same amount of Δ350 SMARCAD1 is required as WT SMARCAD1 to assemble 50% nucleosome product (K_assembly_) (Figs. 5*A*, S7, and Table 2). However, the nucleosome product yield from Δ350 SMARCAD1 is reduced compared to WT SMARCAD1 (Fig. 5*B* and Table 2). This means that while the Δ350 mutant assembles nucleosomes with a similar K_assembly_, less nucleosomes are assembled overall when the N-terminal domain is removed. Despite the low binding affinity for histones, Δ350 SMARCAD1 is capable of reduced nucleosome assembly. Additionally, the low histone binding may be partially compensated for in the assembly assay by the higher concentration of reagents (5x more histones). Furthermore, neither form of Δ350 SMARCAD1 is functional in the histone exchange assay (Figs. 5, *C* and *D*, S9, and Table 2) consistent with reduced binding to DNA and histones. Phosphorylated Δ350 SMARCAD1 produces a small amount of product that migrates as a diffuse band of unknown composition, unlike the defined nucleosome band observed with dephosphorylated WT SMARCAD1 (Fig. S9). Together, this data indicates that even though the ATP motor is intact, the N-terminal region is required for efficient exchange activity and for forming a properly folded nucleosome. As such, deletion of the N-terminal 350 amino acids uncouples ATP hydrolysis from histone removal from DNA.

## Discussion

Dissecting the intricate functions and regulatory mechanisms of chromatin remodelers is crucial for understanding the complexities of genomic maintenance and expression. Particularly, when remodelers have several seemingly incongruent functions, such as human SMARCAD1, it is of interest to determine how these various functions are regulated. SMARCAD1, even as a single subunit remodeler, has the traditional chromatin remodeler function of histone exchange in addition to the chaperone-like activity of *de novo* nucleosome assembly. While ATPase activity (which is triggered by the interaction of SMARCAD1 with nucleosomes or DNA) is required for histone removal from existing nucleosomes, the assembly of nucleosomes does not depend on ATP hydrolysis when free histones are provided.

**Figure 6:**
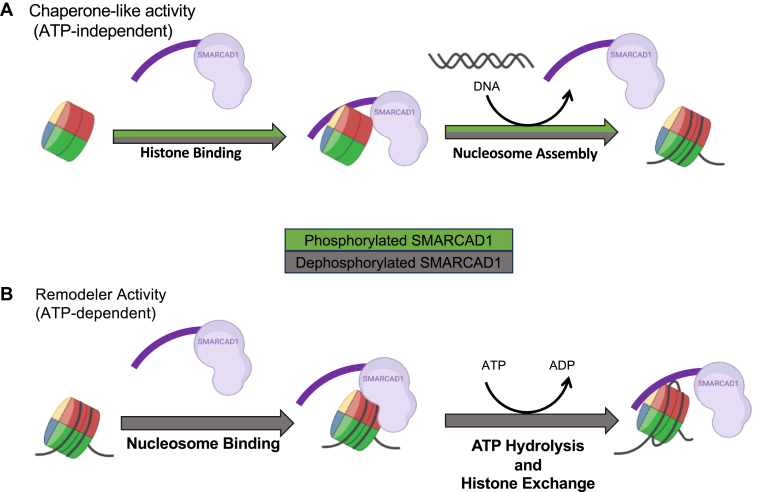
**Working model for the regulation of SMARCAD1 activities.***A*, the ATP-independent chaperone-like activity of SMARCAD1 is not influenced by its phosphorylation state. Both phosphorylated and dephosphorylated forms (represented by *green* and *gray arrows*, respectively) of SMARCAD1 can bind histones and assemble nucleosomes, with the N-terminal region playing a crucial role in these processes. *B*, the ATP-dependent remodeler activity of SMARCAD1 requires dephosphorylation (represented by a *gray arrow*). Dephosphorylated SMARCAD1 binds to nucleosomes and exhibits increased DNA binding, and these interactions are mediated by the C-terminal structured region encompassing the ATPase domain, as supported by structural data (9). SMARCAD1 must be dephosphorylated to hydrolyze ATP in the presence of nucleosomes, or the N-terminal region must be removed. This suggests that the flexible, dephosphorylated N-terminal region (which exhibits an overall pI of 4.2) is required to unwind DNA from the histone bundle. The N-terminal region is also necessary for histone exchange and contributes in a major way to histone binding, implying that SMARCAD1 employs a dephosphorylated N-terminal to remove histones and redeposit them onto another DNA segment to form new nucleosomes.

Data reported here suggest that phosphorylation acts as a regulatory mechanism to switch between the chaperone and chromatin remodeler activities of SMARCAD1 (Fig. 6). The ATP-independent nucleosome assembly activity of SMARCAD1 does not require its dephosphorylation, as both forms of SMARCAD1 can bind histones. In contrast, the removal of histones from an existing nucleosome can only be accomplished by dephosphorylated SMARCAD1. This activity requires that SMARCAD1 interacts with nucleosomes, which in turn is greatly enhanced upon SMARCAD1 dephosphorylation.

Mutation of the previously identified phosphosites in the N-terminal region does not completely abolish the requirement for dephosphorylation to bind nucleosomes. This observation, supported by data presented here and also by proteomics studies in various human benign and cancer cell lines (Fig. S12), suggests that the additional phosphosites outside the N-terminal region are important for SMARCAD1 regulation (20, 21, 22, 23, 24, 25, 26, 27, 28, 29, 30, 31, 32, 33, 34, 35, 36, 37, 38, 39, 40, 41, 42, 43, 44, 45, 46, 47, 48, 49, 50, 51, 52, 53, 54, 55, 56, 57, 58, 59, 60, 61, 62, 63, 64, 65). Importantly, our data suggest that no single specific phosphosite is responsible for inhibiting nucleosome interactions, but rather that there is a phosphorylation threshold beyond which SMARCAD1 is inactive. This global regulation is in addition to the specific phosphorylation sites that contribute to context-dependent SMARCAD1 localization and protein-protein interactions (12, 60, 66, 67, 68), highlighting a complex and layered regulatory framework.

While we show an effect of dephosphorylating SMARCAD1 on its activity, mass spectrometry reveals that residual phosphorylation remain despite CIP treatment (Fig. S13 and Table S1). As such, we expect that our observations between phosphorylated and dephosphorylated SMARCAD1 would be more pronounced upon full dephosphorylation. It is possible that residual phosphosites remain due to their limited accessibility to CIP treatment. Furthermore, the extent and location of phosphorylation appear to be heterogeneous across biological replicates. The source for this heterogeneity is currently unknown. Finally, multiple sequence alignments for mouse, *Xenopus*, *Drosophila*, yeast, *C. elegans*, and human SMARCAD1 shows that phosphosites are not conserved across SMARCAD1 orthologs (Fig. S1*A*). If SMARCAD1 from other species is also regulated by phosphorylation, this speaks to the idea that SMARCAD1 is regulated by a phosphorylation threshold.

We found that SMARCAD1 histone exchange is more active on an undersaturated tri-nucleosome substrate, presumably consisting of a mixture of fully formed nucleosomes and hexasomes. As mass photometry analysis only reveals the overall mass of the tri-nucleosome, the exact composition of each nucleosome is unknown. However, this data suggests that SMARCAD1 prefers some form of sub-nucleosomal substrate for histone exchange. Additionally, when given an oversaturated tri-nucleosome substrate (*i.e.* extra histones associated), SMARCAD1 is active in the absence of ATP. This is consistent with the chaperone-like ATP-independent nucleosome assembly activity observed when SMARCAD1 is provided with free histones (*de novo* assembly).

‘Acidic tails’ are a common feature of histone chaperones. For example, an acidic C-terminal domain that serves as a DNA mimic is essential for the histone chaperone FACT to bind partially unwound nucleosomes, and prevents unwound DNA from re-binding the exposed histone dimer (75). SMARCAD1 has an acidic N-terminal region (residues 1–350; overall pI of 4.2). Our data obtained with the Δ350 SMARCAD1 mutant supports a tentative model where this N-terminal region could be required for prying off histones from nucleosomes and also to efficiently assemble these histones onto DNA to form a *de novo* nucleosome.

Many remodelers are regulated by autoinhibition (69, 76, 77, 78, 79, 80, 81). For example, when the macro domain of the chromatin remodeler ALC1 binds to PARylated PARP, it induces a conformational change that relieves autoinhibition of the ATPase motor, thereby activating ALC1 remodeling at sites of DNA damage (76). The double chromodomains of the Chd1 remodeler block DNA binding and ATPase activity and require binding to the nucleosome acidic patch to release the inhibition and allow for remodeling (78). The ISWI chromatin remodeler contains two autoinhibitory domains (AutoN and NegC) and also requires interactions with the acidic patch of the nucleosome for activation (77). The N-terminal SAM-key in the yeast SMARCAD1 homologue Fun30 binds to the region between the split ATPase domain and acts as an intrinsic, allosteric activator to regulate ATPase activity (70). For SMARCAD1, phosphorylation of the N-terminal region might promote its interaction with the ATPase domains, resulting in its inability to engage with nucleosomes and trigger ATP hydrolysis for histone removal. A version of SMARCAD1 completely lacking the N-terminal domain is still activated by nucleosomes, and, despite its compromised histone binding activity, manages to deposit some onto DNA into non-canonical products. This suggests that there are steps in the histone exchange activity of SMARCAD1 that are yet uncharacterized and speaks to the complex reaction cycle that is characteristic of many remodelers.

## Experimental procedures

### Cloning, expression, and protein purification

We purchased a plasmid containing the coding region of human SMARCAD1 isoform 2 (with a naturally occurring V974A polymorphism) from DNASU (9). This plasmid was polymerase chain reaction (PCR)–amplified to have either a C-terminal or N-terminal 6-His tag. The two versions of purified SMARCAD1 behave the same in binding, nucleosome assembly, and histone exchange assays. We used a primer to PCR amplify the Δ350 SMARCAD1 version. We purchased a plasmid encoding the 23A SMARCAD1 mutant from VectorBuilder, with the indicated 23 serine/threonine to alanine mutations in a pBV Vector system. WT and mutant SMARCAD1 were expressed and purified as described previously (9).

To amplify the virus, plasmids with the SMARCAD1 coding region were used to make a bacmid and then amplified using the Bac-to-Bac expression system. Purified bacmid (≥100 ng/*μ*l) was mixed with Cellfectin and seeded onto a plate containing 3 ml of 0.75 M Sf9 insect cells for 3 days. The supernatant was then added to 0.75 M Sf9 insect cells and grown for 4 days. This step was repeated for another generation. The virus was then titered and virus ≥ 10^8^ was added to 300 ml of Sf9 cells at MOI = 1 and expressed for 4 days until cells swelled to at least 12 *μ*m.

For purification, cells were resuspended in lysis buffer (250 mM NaCl, 20 mM HEPES (pH 7.5), 2 mM Tris (2-carboxyethyl) phosphine, 1 mM 4-(2-aminoethyl) benzenesulfonyl fluoride hydrochloride, 10% glycerol, and 2 mM MgCl_2_) supplemented with a cOmplete Protease Inhibitor Cocktail (Roche Diagnostics) and 1500 U of Benzonase (Millipore Sigma) per 300 ml of Sf9 insect cells. For Δ350 SMARCAD1, the lysis buffer contained 1 M NaCl to ensure the truncated protein did not bind to DNA. Cells were lysed using a TissueLyser (Tekmar), sonicated, and spun at 31,000 RCF (Beckman JA-20 rotor) for 30 min. The supernatant was then filtered using a 0.45 *μ*m nylon membrane filter (Millipore Sigma) and purified over a 5-mL nickel column and a 5-mL HiTrap-Q column (Cytiva Life Sciences).

The sample was split for phosphatase treatment to prepare dephosphorylated protein; ‘mock phosphatase treatment’ was performed in parallel for the phosphorylated samples. To dephosphorylate SMARCAD1, 500 U of Quick Calf Intestinal Alkaline Phosphatase (CIP) (New England BioLabs) were added per mg SMARCAD1 and rotated for 1 h at room temperature. Both the phosphorylated and dephosphorylated samples were then separately purified over a 1 ml nickel column, followed by S200 size exclusion chromatography (GE) in S200 buffer (100 mM KCl, 20 mM HEPES (pH 7.5), 2 mM TCEP). Samples were stored at −80 °C in S200 buffer, supplemented with 10% glycerol.

### Digested mass spectrometry and identification of phosphorylation sites

Purified WT SMARCAD1, 23A SMARCAD1, and Δ350 SMARCAD1 (∼20 *μ*g each) were prepared using the Single-Pot Solid-Phase-enhanced Sample Preparation (SP3) method and analyzed in the mass spectrometry core facility at the University of Colorado Boulder (82). The protein was denatured in 5% (wt/vol) sodium dodecyl sulfate, 50 mM Tris-HCl pH 8.5, 80 mM 2-chloroacetamine, 20 mM Tris (2-carboxyethyl) phosphine hydrochloride (TCEP-HCl). Samples were boiled at 95 °C for 10 min, shaken at 200 rpm for 30 min at 37 °C, sonicated, and added to 0.1 mg/ml carboxylate-functionalized speedbeads (GE Life Sciences). Next, 80% acetonitrile (vol/vol) was added to the sample to precipitate the protein and allow it to bind to the beads. The sample was washed twice with 80% (vol/vol) ethanol and again with 100% acetonitrile. The sample was then incubated at 37 °C overnight with 1:50 LysC/Trypsin (Promega) to protein ratio in 50 mM Tris pH 8.5. Next, the sample was cleaned using the Water Oasis HLB 1 cc (10 mg) cartridge, speed-vac rotary evaporated and resuspended in 0.1% trifluoroacetic acid (TFA), 3% acetonitrile in water to peptides on a rpC18 column for liquid chromatography/MS (LC/MS) analyses. Eluted peptides were then collected and injected onto a Waters M-class column (1.7 *μ*m, 120A, rpC18, 75 *μ*m × 250 mm) and eluted from a 2% to 20% acetonitrile gradient for 40 min at 0.3 *μ*l/min using a Thermo Ultimate 3000 ultra performance liquid chromatography (UPLC) (Thermo Scientific). The peptides were detected using the Thermo Q-Exactive HF-X mass spectrometer (Thermo Scientific). Raw files were searched in the Max-Quant software (MaxQuant) using a SMARCAD1 FASTA file and *Spodoptera frugiperda* SF9 Insect Cell Line FASTA file. Phosphorylation sites were detected with the phosphorylation (STY) modification variable modification setting on MaxQuant.

### Intact mass spectrometry

For intact protein analyses, proteins were directly injected onto a 2.1 × 5 mm Acquity UPLC BEH300 C4, 1.7 *μ*m VanGuard Pre-Column (Waters), using a Waters Acquity classic UPLC. Protein were loaded and washed at 0.2 ml/minute for 3 min, then eluted with a gradient from 3% to 85% ACN in 3 min then to 95% ACN in 0.5 min and detected using a Synapt G2 Q-Tof mass spectrometer (Waters). Precursor mass spectra (MS1) were acquired in positive ES resolution mode from 200 to 2500 m/z with 3.0 kV capillary voltage and a source temperature of 80 °C. Intact protein masses were deconvoluted using Mass Lynx v4.2 Maximum Entropy.

### Alphafold 3D prediction

The predicted 3D model for SMARCAD1 (AlphaFold 3) (83) was visualized using PyMol v2.5.4 (PyMOL Molecular Graphics System, Version 1.2, Schrödinger, LLC).

### Histone refolding and nucleosome reconstitution

We purchased human histones in lyophilized form from the Histone Source at Colorado State University (Fort Collins, CO). Histones were refolded into either H2A-H2B dimer, (H3-H4)_2_ tetramer, or octamer as described (84). For fluorescent assays, either H2B was labeled at T115C with maleimide-Atto 647, or H4 was labeled at E63C with maleimide-Alexa Fluor 488, as stated in the text (85). 165 bp DNA containing the 147 bp Widom 601 nucleosome positioning sequence (underlined below) flanked by 7 bp and 11 bp on either side was purified as described previously (84). Nucleosomes were reconstituted using the salt gradient method (84).

CGAGCCAGGCCTGAGAATCCGGTGCCGAGGCCGCTCAATTGGTCGTAGACAGCTCTAGCACCGCTTAAACGCACGTACGCGCTGTCCCCCGCGTTTTAACCGCCAAGGGGATTACTCCCTAGTCTCCAGGCACGTGTCAGATATATACATCCAGGCCTTGTGTCG.

### Fluorescence polarization binding assay

To monitor SMARCAD1-nucleosome binding, we reconstituted a 165 bp DNA fragment (sequence listed above) with histone octamer, using histone H4 labeled with Alexa Fluor 488 at E63C. To test SMARCAD1-DNA binding, we used an Alexa Fluor 488 labeled primer (Integrated DNA Technologies) and an Alexa Fluor 647 labeled primer (Integrated DNA Technologies) to PCR-amplify a 165 bp DNA fragment (same sequence as above). In both binding experiments, we added increasing concentrations of SMARCAD1 (0–5 μM) to labeled nucleosomes or DNA (10 nM) in nucleosome/DNA FP buffer (2 mM DTT, 10% glycerol, 50 mM HEPES (pH 7.5), 0.01% CHAPS, 0.01% NP-40). Samples were incubated at room temperature for 30 min and then the change in FP signal was record in a BMG Labtech CLARIOstar plate reader. A well containing only labeled DNA/nucleosome was set at an FP value of 200 mP and binding constants were calculated using a hyperbolic binding equation (Equation 1):

(1) $$Y=Y_{\min}+\left(Y_{\max}\;-{\;Y}_{\min}\right)\times \left[\text{SMARCAD}1\right]/\left(K_{D}+\left[\text{SMARCAD}1\right]\right)$$

To monitor SMARCAD1-histone binding, 5 nM of H2A-H2B dimer (fluorescently labeled with Alexa Fluor 488 on H2B T115C) or 5 nM of (H3-H4)_2_ tetramer (fluorescently labeled with Alexa Fluor 488 on H4 at E63C) was combined with increasing concentrations of SMARCAD1 (0–2500 nM) in histone FP buffer (2 mM DTT, 10% glycerol, 50 mM HEPES (pH 7.5), 0.01% CHAPS, 0.01% NP-40, and 300 mM KCl). Samples were incubated at room temperature for 30 min and then the change in FP signal was recorded in a BMG Labtech CLARIOstar plate reader. A well with only H2A-H2B or H3-H4, respectively, was set at an FP value of 200 mP and binding constants were calculated using a hyperbolic binding equation (Equation 1).

### ATP hydrolysis assay

To measure the ability for SMARCAD1 to hydrolyze ATP in the presence of either DNA or nucleosome activator, we added lactate dehydrogenase (LDH) and pyruvate kinase (PK) to oxidize NADH as a function of ATP hydrolysis, indicted as a decrease in A340 signal. This reaction was done in ATP hydrolysis buffer (1 mM DTT, 4 mM MgCl_2_, 1 mM phosphoenolpyruvate, and 12 μl LDH/PK enzyme mix (Sigma-Aldrich), 50 mM HEPES (pH 7.5), 100 mM KCl, and 0.7 mM NADH). Reactions were conducted by mixing a 1:1 ratio of SMARCAD1 (1 *μ*M) to activator (1 *μ*M DNA or 1 *μ*M nucleosome). The reactions were initiated by addition of 1 mM ATP and the change in A340 signal was monitored in a BMG Labtech CLARIOstar plate reader. Next, the slope of the change of A340 was obtained by linear regression and the change in A340/s was converted to ATP hydrolyzed (M/s) using the NADH extinction coefficient (6330 M^−1^cm^−1^). A buffer-only control was subtracted from all samples and rates were recorded as change in M/s.

Percent saturation values under ATPase assay conditions were determined using the quadratic equation (Equation 2):

(2) $$\text{Percent}\;\text{Saturation}=100*(\left(\text{Kd}+\left[\text{SMARCAD}1\right]+\left[\text{activator}\right]\right)-\text{SQRT}\left(\left(\text{Kd}+\left[\text{SMARCAD}1\right]+\left[\text{activator}\right])ˆ2\;-\;4*\left[\text{SMARCAD}1\right]*\left[\text{activator}\right]\right)\right)/\left(2*\left[\text{SMARCAD}1\right]\right)$$

where both [SMARCAD1] and [activator] are 1 uM and Kd values as shown Table 1. For P WT and P Δ350 nucleosome data, Kd values were estimated by forcing a global upper plateau to the binding data, therefore, the calculated % saturation represents an estimated maximum value and is reported as such, <29% and <42%, respectively.

### *De novo* nucleosome assembly assay

SMARCAD1 (3 *μ*M) was mixed with histone octamer (Atto647N H2BT115C; 50 nM) for 15 min in assembly buffer (2 mM DTT, 100 mM KCl, 50 mM HEPES (pH 7.5), 10% glycerol, and 2 mM MgCl_2_). Next, Widom 147-bp DNA (50 nM, underlined sequence above) was added and incubated at room temperature for 15 min. Samples were then quenched with 16% glycerol, 68 mM EDTA, and 2.7 μg of pUC19 plasmid (to compete off SMARCAD1 from the nucleosome product) and run on a 5% native TBE gel. The gel was then imaged on a Typhoon imager in fluorescence mode. Analysis of the imaged gel was completed by quantifying the bands using ImageQuant software, plotted and fit using Prism. Nucleosome product formation was normalized to dephosphorylated WT product and then plotted as a function of increasing SMARCAD1 concentration, curves were then fit to (Equation 3):

(3) Y=Vmax∗X/(K+X)

Here, the K value represents the amount of SMARCAD1 required to assemble 50% nucleosome product (here, termed the K_assembly_).

### Histone exchange assay

Tri-nucleosomes with histone H2B labeled at T115C with Widom 601 positioning sequencing and flanking DNA (30N60N60N30; N denotes a nucleosome) were assembled as described (84). SMARCAD1 (3 *μ*M) was added to tri-nucleosomes (62.5 nM) and 1.5 *μ*M acceptor DNA (Widom 601, 147 bp DNA) in 2 mM DTT, 2 mM MgCl_2_, 25 mM HEPES (pH 7.5), and 50 mM KCl. Reactions were initiated with addition of 1 mM ATP, incubated at 30 °C, and then quenched with quench buffer (68 mM EDTA and 16% glycerol, final concentration) at 3, 7, 15, 30, and 40 min timepoints. Samples were then run on a 5% TBE gels and imaged for 647 fluorescence on the Typhoon imager. Analysis of the imaged gel was completed by quantifying the bands using ImageQuant software, plotted and fit using Prism. To quantitatively determine the rate values, time courses were fit to the following equation for one-phase association equation (Equation 4):

(4) $$Y=Y_{0}+\left(\text{Plateau}\;–\;Y_{0}\right)\times \left(1\;-\exp\left(–K\times x\right)\right)$$

### Mass photometry

Mass photometry measurements were performed on a Refeyn TwoMP mass photometer (Refeyn Ltd). Glass coverslips were first cleaned with isopropanol, deionized water, and dried with N_2_ gas, before being coated with a 0.01% Poly-L-Lysine solution for 20 s, rinsed with water, and dried with N_2_ gas. To form a sample chamber, self-adhesive silicon gaskets were adhered to the top of the treated coverslip. For each measurement, the coverslip was placed on the oil-immersion objective lens, centered on a single well, and 13.5 *μ*l of sample buffer (20 mM HEPES, pH 7.5, 100 mM KCl) was added to the well and the focal position of the glass surface was determined and held constant using an autofocus system. Samples were first diluted to 100 to 200 nM, before a final 10-fold dilution onto the sample stage (final concentration of 10–20 nM). All dilutions were performed at room temperature in sample buffer (20 mM HEPES, pH 7.5, 100 mM KCl). A 60 s video was recorded immediately after the final dilution. A fresh well and dilution was used for each measurement and repeated at least three times for each sample. Tri-nucleosomes were diluted in buffer so that the number of detected events (particle counts) during the 60 s measurement was roughly 4000 to 9000 for an optimum data acquisition and processing. A known mass standard (β-amylase and thyroglobulin) was used to convert image contrast-signal into mass units. To calculate the molecular weight of the main species observed on the particle counts *versus* molecular mass distribution histograms we used the Gaussian function in the DiscoverMP software.

## Data availability

The mass spectrometry proteomics data have been deposited to the ProteomeXchange Consortium *via* the PRIDE (86) partner repository with the dataset identifiers PXD055412 and 10.6019/PXD055412; PXD055413 and 10.6019/PXD055413; PXD055414 and 10.6019/PXD055414; and PXD055450 and 10.6019/PXD055450.

## Supporting information

This article contains supporting information.

## Conflicts of interest

The authors declare that they have no conflicts of interest with the contents of this article.
