# Supplementary material for: Phosphorylation regulates the chromatin remodeler SMARCAD1 in nucleosome binding, ATP hydrolysis, and histone exchange[image]
Source: J Biol Chem. 2024 Oct 17;300(12):107893. doi: 10.1016/j.jbc.2024.107893 (PMC11742319; doi:10.1016/j.jbc.2024.107893)
Supplement: Supporting information [file mmc1.pdf]

SUPPORTING INFORMATION

Phosphorylation regulates the chromatin remodeler SMARCAD1 in nucleosome binding, ATP-hydrolysis, and histone exchange

Briana L. Aboulache[1,2], Nicole M. Hoitsma[1,2], Karolin Luger[1,2]*

[1] Department of Biochemistry, University of Colorado Boulder, 80309 Boulder, Colorado

[2] Howard Hughes Medical Institute, Chevy Chase, Maryland

*Corresponding author: karolin.luger@colorado.edu

Figure S1. Multiple sequence alignment of SMARCAD1

Figure S2. SMARCAD1 purification scheme

Figure S3. Native gel of SMARCAD1 with nucleosome

Figure S4. Native gel of SMARCAD1 with DNA

Figure S5. Fluorescence Polarization of histone binding

Figure S6. Raw change in A340/sec for SMARCAD1 ATP hydrolysis

Figure S7. Native gel of SMARCAD1 nucleosome assembly

Figure S8. Optimization of tri-nucleosome substrate for SMARCAD1 histone exchange

Figure S9. Native gel of SMARCAD1 histone exchange

Figure S10. Alphafold3 structure prediction of SMARCAD1

Figure S11. Intact mass spectrometry of Δ350 SMARCAD1

Figure S12. Phosphoproteomic publications of human SMARCAD1

Figure S13. Mass spectrometry reveals varying levels of phosphorylation

Table S1. The percent phosphorylation of all WT SMARCAD1 peptides

A

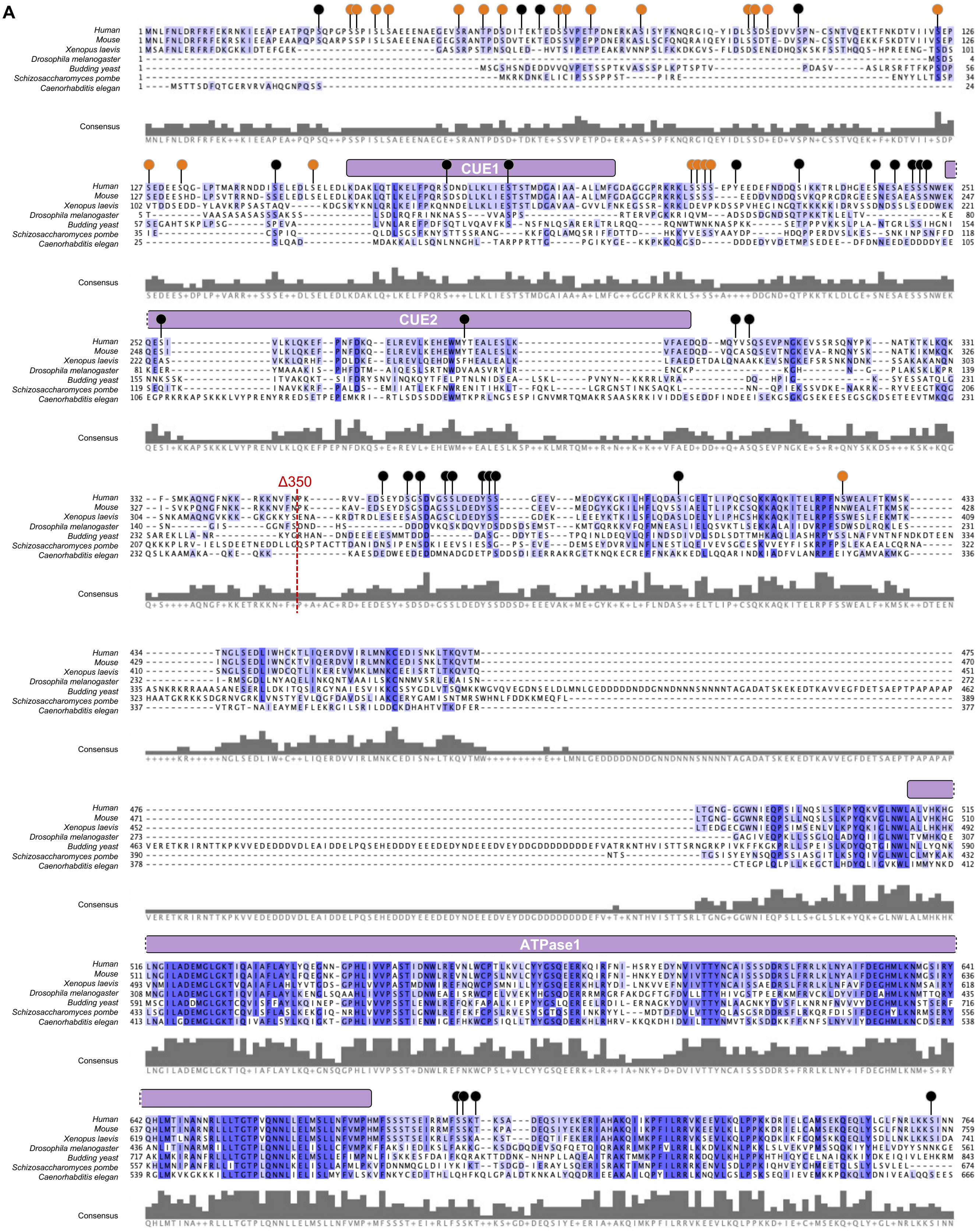

Human
Mouse
Xenopus laevis
Drosophila melanogaster
Budding yeast
Schizosaccharomyces pombe
Caenorhabditis elegan
Consensus
CUE1
CUE2
Δ350
ATPase1

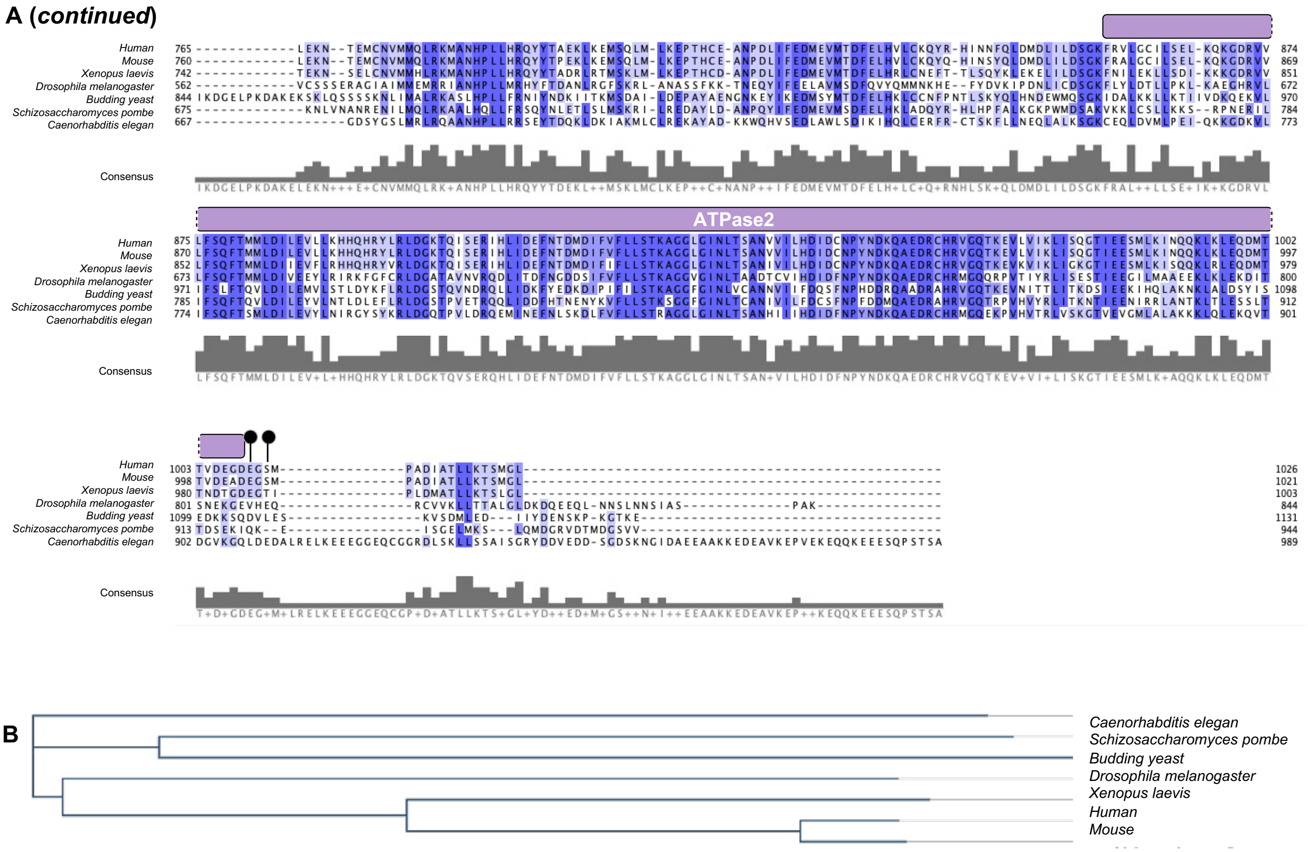

Figure S1. Multiple sequence alignment of SMARCAD1

(**A**) Multiple sequence alignment of WT SMARCAD1 from a diverse set of model organisms. Sequences were aligned using the ClustalWS algorithm within the Jalview software (v2.11.0). Domains are indicated with purple boxes and phosphorylated residues are shown as circles, with previously reported phosphosites in orange (ref. 9) and additional phosphosites identified in WT SMARCAD1, 23A SMARCAD1, and Δ350 in this study in black.

(**B**) A tree of SMARCAD1 orthologs showing the relationship of species used in the multiple sequence alignment in ClustalO UniProt software (v1.2.4).

A

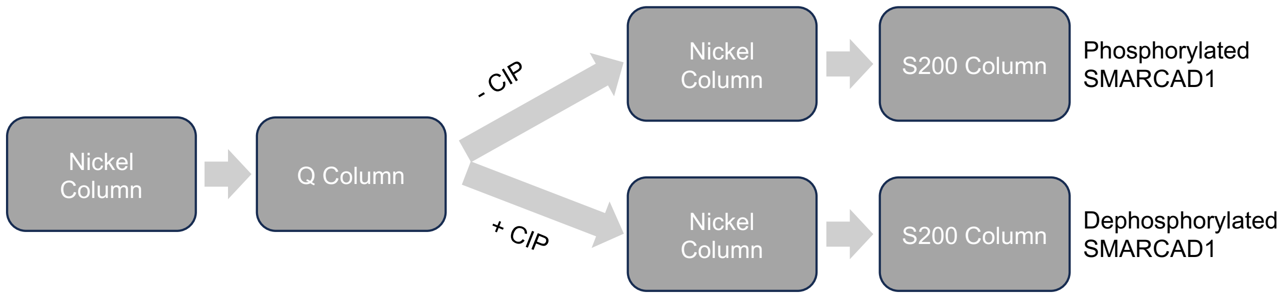

B

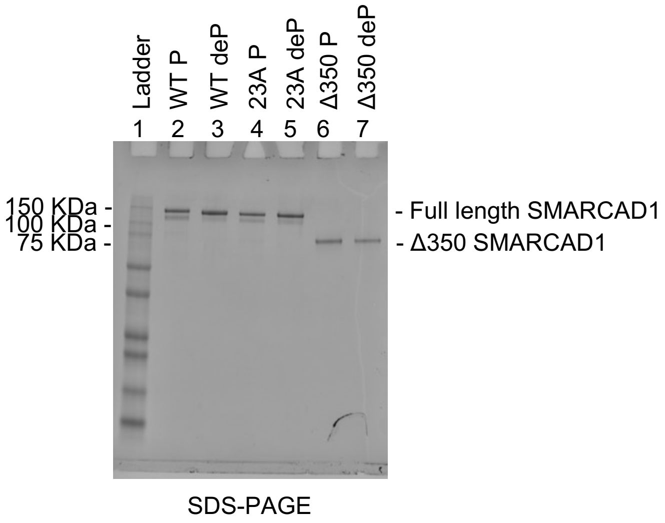

Figure S2. SMARCAD1 purification scheme

(**A**) Purification scheme for phosphorylated and dephosphorylated SMARCAD1 expressed in Sf9 insect cells. Protein was purified over a 5 mL nickel column, a 5 mL Q column, then split into phosphorylated and dephosphorylated (+/- Quick Calf Intestinal Alkaline Phosphatase) samples, dephosphorylated for an hour at room temperature, loaded onto a 1 mL nickel column, and run on a SEC S200 column.

(**B**) SDS-PAGE of purified phosphorylated and dephosphorylated WT SMARCAD1 (118 KDa), 23A SMARCAD1 (118 KDa), and Δ350 SMARCAD1 (78 KDa).

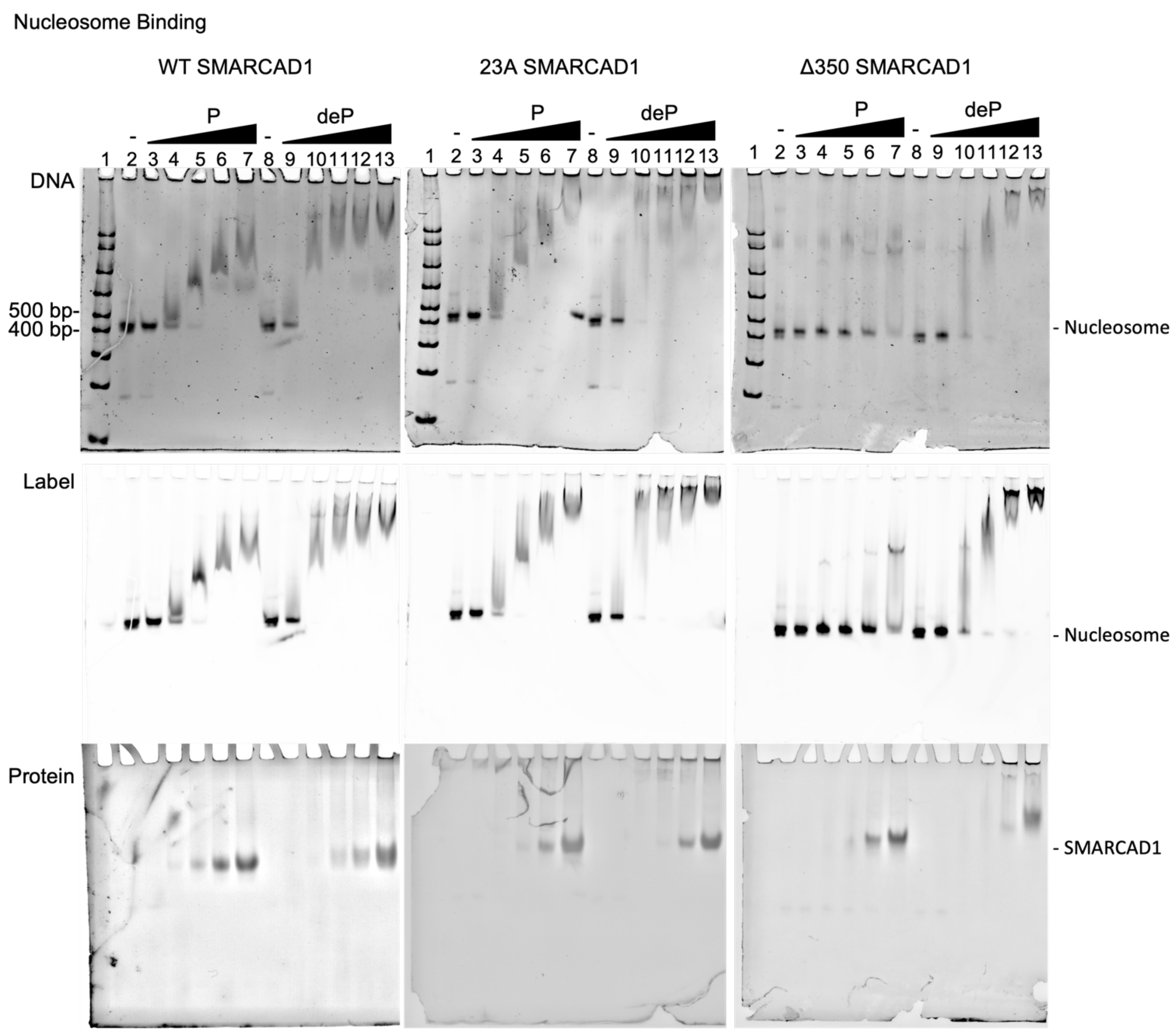

Figure S3. Native gel of SMARCAD1 with nucleosome

Electrophoretic Mobility Shift Assay (EMSA) of WT SMARCAD1, 23A SMARCAD1, and Δ350 SMARCAD1 (0, 0.01, 0.08, 0.31, 1.25, and 5 μM) in phosphorylated (P) and dephosphorylated (deP) forms, with Alexa Fluor 488–labeled 7N11 nucleosome (10 nM), analyzed on a 5% native TBE gel. SMARCAD1-nucleosome binding is indicated by an up-shift of the nucleosome band. The same gel was imaged using three different settings: EtBr to visualize DNA (top), Alexa Fluor 488-label (middle), Instant Stain to visualize protein (bottom).

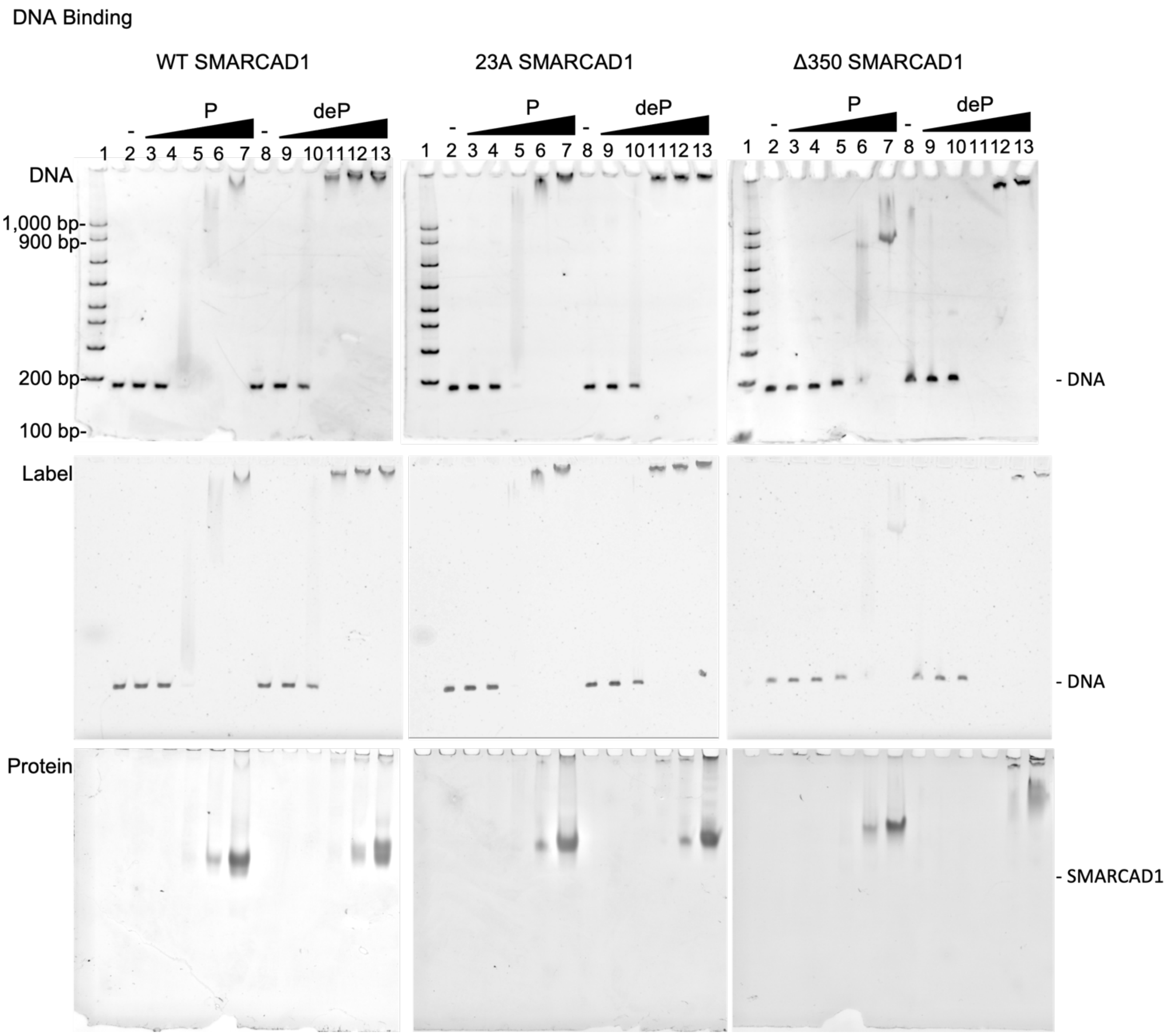

Figure S4. Native gel of SMARCAD1 with DNA

EMSA of WT SMARCAD1, 23A SMARCAD1, and Δ350 SMARCAD1 (0, 0.001, 0.01, 0.08, 0.63, and 5 μM) in phosphorylated (P) and dephosphorylated (deP) forms with Alexa Fluor 488–labeled 165 DNA (10 nM) ran on a 5% native TBE gel. SMARCAD1-DNA binding is indicated by a shift up of the DNA band. The same gel was imaged using three different settings: EtBr to visualize DNA (top), Alexa Fluor 488-label (middle), Instant Stain to visualize protein (bottom).

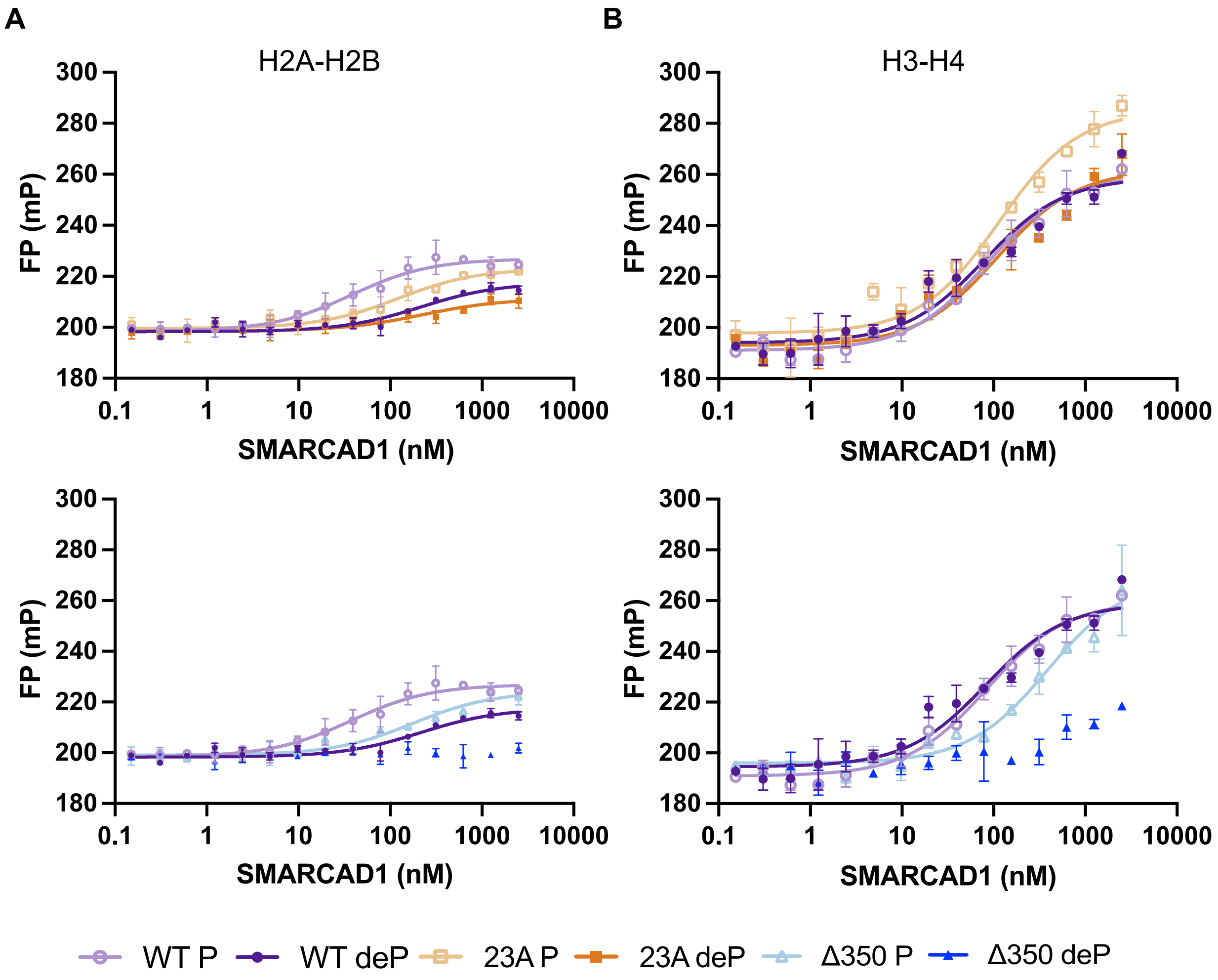

Figure S5. Fluorescence Polarization of histone binding

(**A**) WT, 23A, and Δ350 SMARCAD1 in phosphorylated (P) and dephosphorylated (deP) forms with histone H2A-H2B (Alexa Fluor 488-H2B, 5 nM), shown as one representative curve (error bars represent technical duplicate).

(**B**) WT, 23A, and Δ350 SMARCAD1 in phosphorylated (P) and dephosphorylated (deP) forms with histone H3-H4 (Alexa Fluor 488-H4, 5 nM), shown as one representative curve (error bars represent technical duplicate).

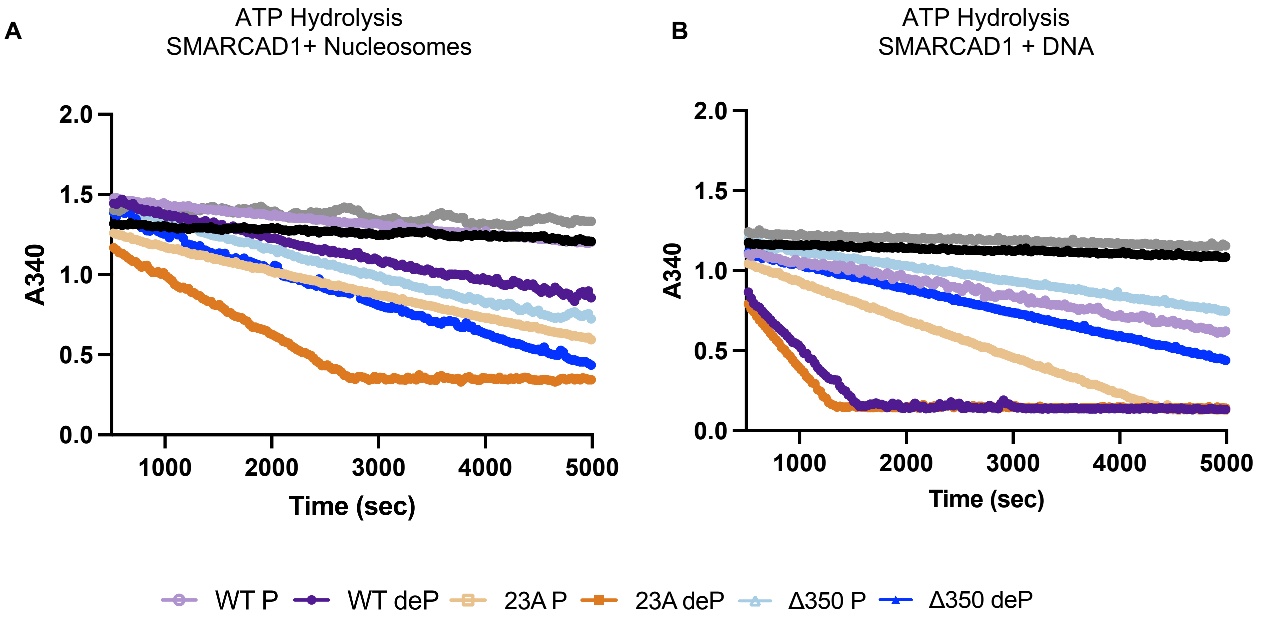

Figure S6. Raw change in A340/sec for SMARCAD1 ATP hydrolysis

Change in A340/sec for ATP Hydrolysis from 500 – 5000 sec. SMARCAD1 (1 μM) was mixed with (**A**) 7N11 nucleosome (1 μM) or (**B**) 165 bp DNA (1 μM). The rate of ATP hydrolysis per second was coupled to pyruvate kinase and lactose dehydrogenase metabolic enzymes which ultimately results in the oxidation of NADH and thus a decrease in A340 absorbance. Only the initial linear portions were quantified.

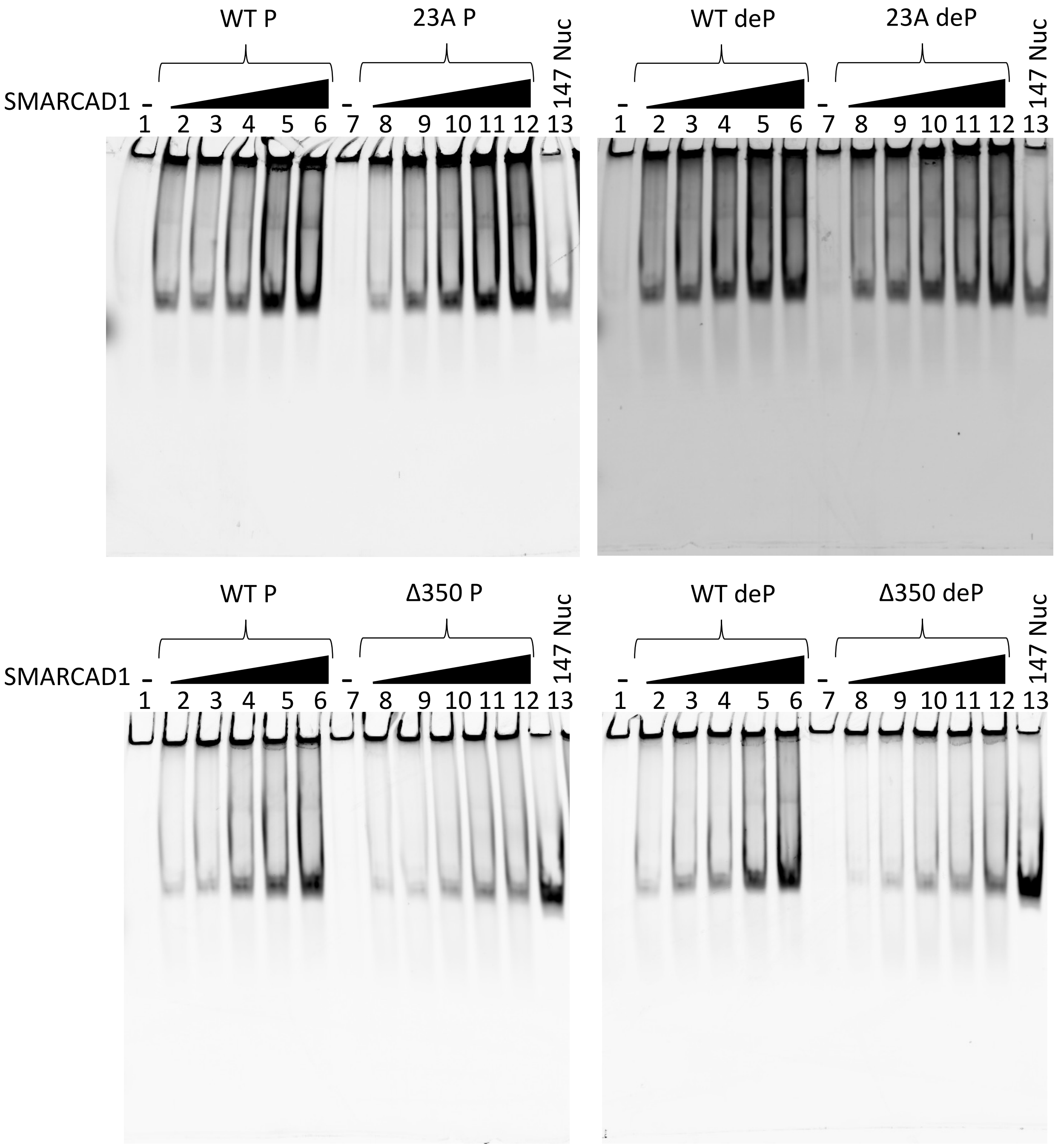

Figure S7. Native gel of SMARCAD1 nucleosome assembly

Five percent native TBE gel of *de novo* nucleosome assembly from histones and DNA. The gel was imaged for 647 label. SMARCAD1 concentrations were 0, 0.2, 0.4, 0.8, 1.5, and 3 μM.

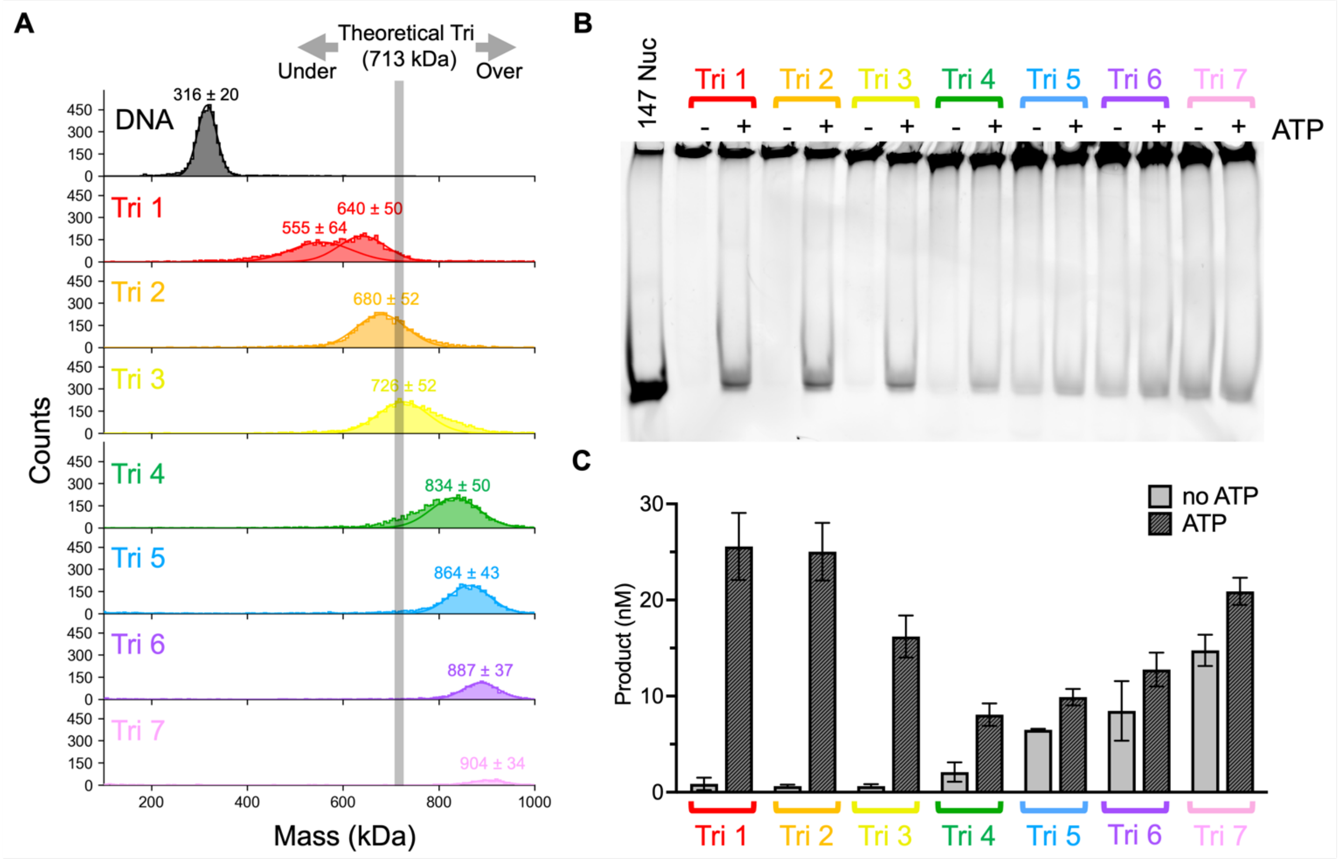

Figure S8. Optimization of tri-nucleosome substrate for SMARCAD1 histone exchange

(**A**) Mass photometry analysis of tri-nucleosome substrates; DNA (black, top) with tri-nucleosomes arranged from undersaturated (red, orange) to oversaturated (green, blue, purple, pink). The solid lines represent the Gaussian function fit to the main species observed on particle counts versus molecular mass distribution histograms, with the estimated molecular weight (in kDa) corresponding to the respective mass at the center of each peak (± SD).

(**B**) Representative gel (5% TBE native) of SMARCAD1 histone exchange assay with each of the tri-nucleosome substrates with

(**C**) quantification of the nucleosome product band (n=3). Here, all reactions were quenched at 40 minutes and quantified relative to the 147 nucleosome control.

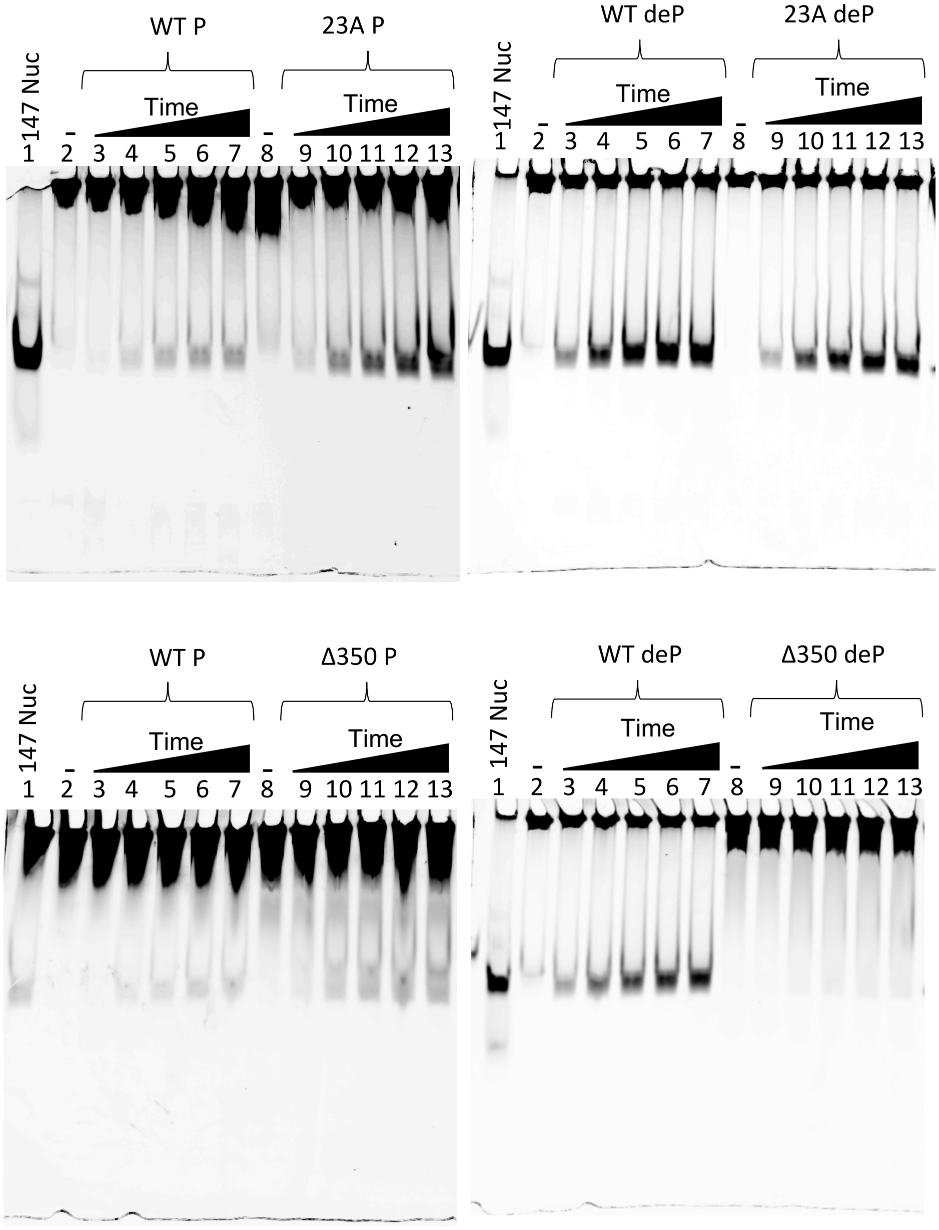

Figure S9. Native gel of SMARCAD1 histone exchange

Five percent TBE gel showing histone exchange of WT SMARCAD1, 23A SMARCAD1, or Δ350 SMARCAD1 (3 μM). SMARCAD1 removes Atto647-labeled H2A-H2B histones from 30N60N60N30 tri-nucleosomes (75 nM) and assembles them onto a piece of 147 DNA (1.5 μM) to form a product that runs the same as a mono-nucleosome control. The reaction was quenched at varying timepoints (3, 7.5, 15, 30, and 40 min) and run on a 5% native TBE gel and the Atto647 labeled mono-nucleosome formation was quantified. (-) indicates no ATP was added.

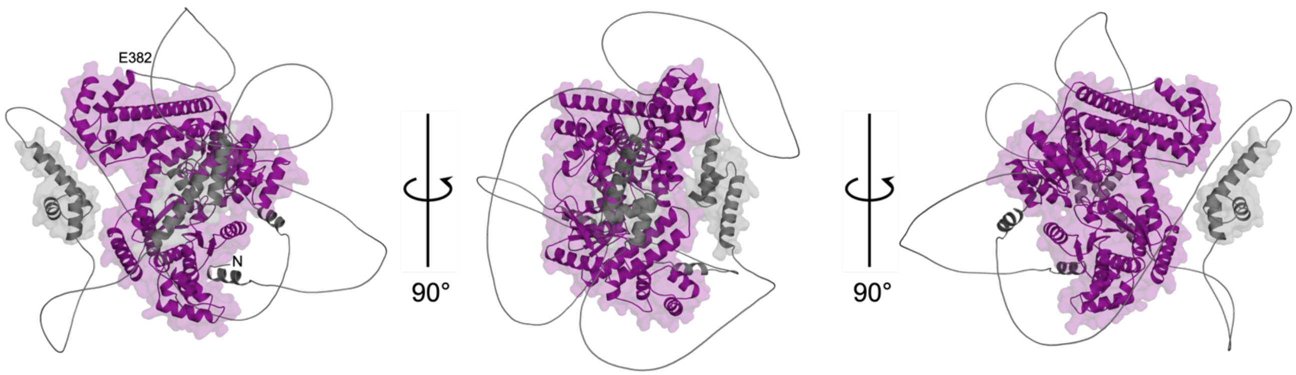

Figure S10. Alphafold3 structure prediction of SMARCAD1

The N-terminal region is mostly unstructured with the exception of the two CUE domains (residues 1-382, shown as grey cartoon with the structured CUE domains with gray surface). The globular C-terminal region (residues 383-1026) is shown as purple cartoon and surface representation.

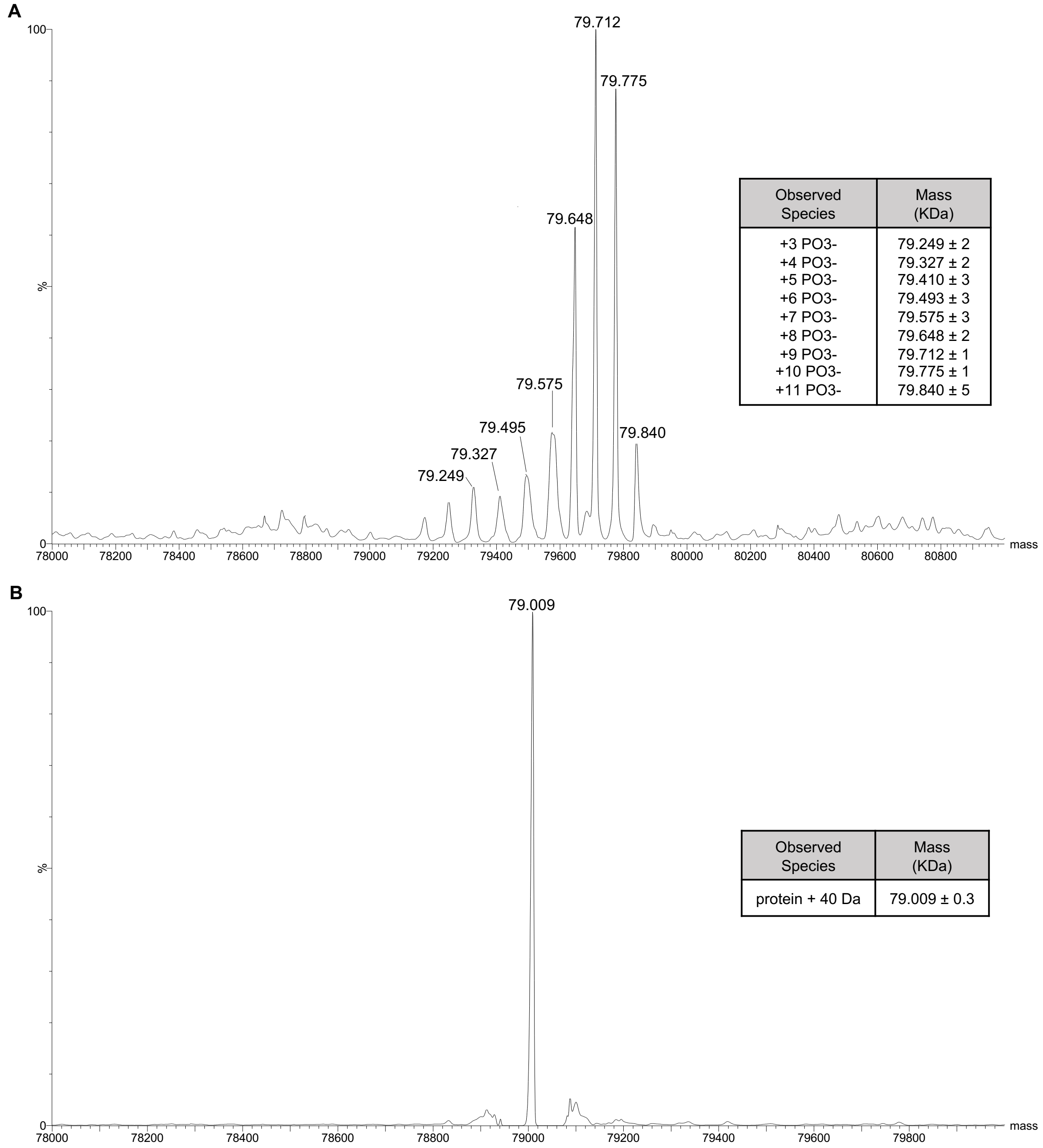

Observed Species	Mass (KDa)
+3 PO3-	79.249 ± 2
+4 PO3-	79.327 ± 2
+5 PO3-	79.410 ± 3
+6 PO3-	79.493 ± 3
+7 PO3-	79.575 ± 3
+8 PO3-	79.648 ± 2
+9 PO3-	79.712 ± 1
+10 PO3-	79.775 ± 1
+11 PO3-	79.840 ± 5

Observed Species	Mass (KDa)
protein + 40 Da	79.009 ± 0.3

Figure S11. Intact mass spectrometry of Δ350 SMARCAD1

(**A**) Intact mass spectrometry of phosphorylated Δ350 SMARCAD1 shows 3-11 phosphate groups.

(**B**) Intact mass spectrometry of dephosphorylated Δ350 SMARCAD1 shows only one peak, indicating a single species of acetylated protein without any phosphate groups.

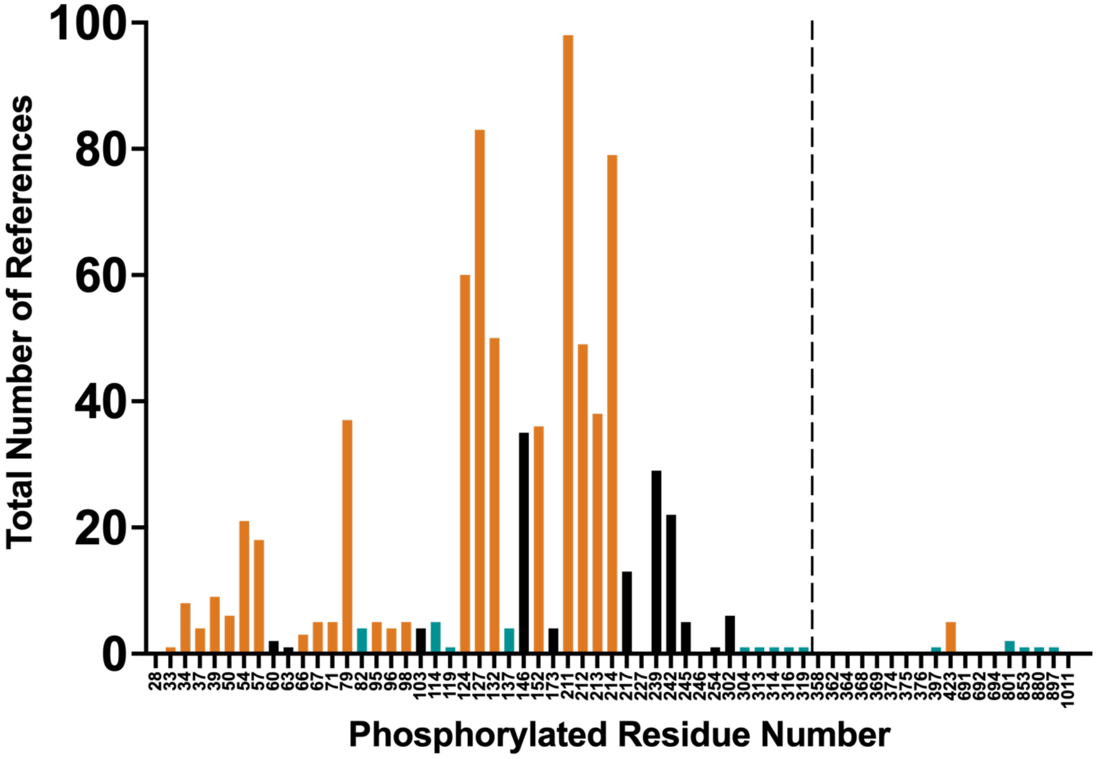

Figure S12. Phosphoproteomic publications of human SMARCAD1

All phosphorylation sites found on human SMARCAD1 (in human cells and via insect cell expression) are shown on the x-axis, bars represent number of times that phosphosite has been reported from human cells studies in either journal publications or the cell signaling technology research group (20-65). These high throughput papers used epithelial, stem, and blood cell lines in benign and cancer forms. The phosphosites that have been identified for human SMARCAD1 purified from insect cells are shown in orange (phosphosites reported in ref 9, those mutated to alanine in the 23A mutant) and black (additional 26 phosphosites identified in this study). Of the 26 phosphosites identified in this study, 11 have been reported in these human cell studies (shown as black bars), those that have not been reported do not have a bar. There are also 14 phosphosites reported in human cell studies that have not been identified via insect cell expression (shown in cyan) (22, 23, 25). Dashed line shown for reference to which residues are truncated in the Δ350 SMARCAD1 mutant.

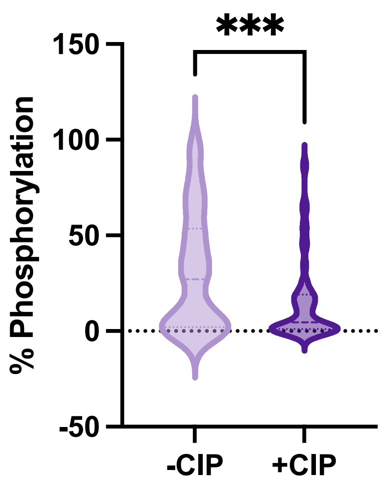

Figure S13. Mass spectrometry reveals varying levels of phosphorylation

Digested mass spectrometry reveals an overall decrease in phosphorylation for phosphatase treated WT SMARCAD1. Quantification of the percent phosphorylation of each peptide with and without CIP-treatment. Peptides are counted as phosphorylated if any combination of its phosphorylation sites were phosphorylated and reported values are mean ± SD of the number of replicates that contained that peptide (n) as shown in Table S1. ***P ≤ 0.001; unpaired *t* test.

Table S1		(- CIP)		(+ CIP)	
Peptide	Phosphorylation Sites	% Phosphorylation	n	% Phosphorylation	n
ANTPDSDITEK	T54, S57	68 ± 22	5	64 ± 28	5
ANTPDSDITEKTEDSSVPETPDNER	T54, S57, T60, T63, S66, S67, T71	97 ± 2	3	86 ± 11	3
ANTPDSDITEKTEDSSVPETPDNERK	T54, S57, T60, T63, S66, S67, T71	32 ± 43	4	87 ± 5	4
ASISYFK	S79	2 ± 0	2	0 ± 0	2
ASISYFKNQR	S79	7 ± 7	3	0 ± 0	3
DTVIIVSEPSEDEESQGLPTM(Oxidation (M))AR	S124, S127, S132	80 ± 16	5	25 ± 18	5
DTVIIVSEPSEDEESQGLPTM(Oxidation (M))ARR	S124, S127	96 ± 0	1	0 ± 0	
DTVIIVSEPSEDEESQGLPTMAR	S124, S127, S132	50 ± 28	5	14 ± 18	5
DTVIIVSEPSEDEESQGLPTMARR	S124, S127	42 ± 2	2	0 ± 0	2
ELFPQRSDNDLLK	S173	18 ± 35	4	57 ± 24	4
EVLKEHEWM(Oxidation (M))YTEALESLK	Y282, T283	0 ± 0	2	0 ± 0	2
EVLKEHEWMYTEALESLK	Y282, T283	0 ± 0	3	0 ± 0	3
GIQYIDLSSDSEDVVSPNCSNTVQEK	S95, S96, S98, S103	32 ± 15	5	5 ± 3	5
IEEAPEATPQPSQPGPSSPISLSAEEENAEGEVSR	S28, S33, S34, S39	46 ± 36	5	19 ± 26	5
ILHFLQDASIGELTLIPQCSQK	-	0 ± 0	1	0 ± 0	1
ITELRPFNSWEALFTK	S423	1 ± 1	5	2 ± 2	4
KASISYFK	S79	1 ± 1	4	9 ± 10	4
KASISYFKNQR	S79	4 ± 5	2	17 ± 25	2
KLSSSSEPYEEDEFNDDQSIK	S211, S212, S213, S214	80 ± 18	4	25 ± 24	4
KLSSSSEPYEEDEFNDDQSIKK	S211, S212, S213, S214, Y217, S227	76 ± 43	5	45 ± 19	5
KTRLDHGEESNESAESSSNWEK	S239, S242, S245, S246, S247	67 ± 30	4	44 ± 31	4
LDHGEESNESAESSSNWEK	S239, S242, S245, S246, S247	27 ± 26	5	15 ± 10	5
LDHGEESNESAESSSNWEKQESIVLK	S239, S242, S245, S254	30 ± 29	3	5 ± 9	3
LDHGEESNESAESSSNWEKQESIVLKLQK	S183, S239, S242	76 ± 22	3	4 ± 6	3
LEQDM(Oxidation (M))TTVDEGDEGSM(Oxidation (M))PADIATLLK	S1011	15 ± 0	1	0 ± 0	1
LEQDM(Oxidation (M))TTVDEGDEGSMPADIATLLK	S1011	2 ± 0	1	0 ± 0	1
LEQDMTTVDEGDEGSM(Oxidation (M))PADIATLLK	S1011	2 ± 0	1	0 ± 0	1
LEQDMTTVDEGDEGSMPADIATLLK	S1011	26 ± 0	1	0 ± 0	1
LIESTSTM(Oxidation (M))DGAIAAALLM(Oxidation (M))FGDAGGGPR	S183	0 ± 0	1	21 ± 0	1
LIESTSTM(Oxidation (M))DGAIAAALLMFGDAGGGPR	S183	0 ± 0	1	1 ± 0	1
LIESTSTMDGAIAAALLM(Oxidation (M))FGDAGGGPR	S183	0 ± 0	1	2 ± 0	1
LIESTSTMDGAIAAALLMFGDAGGGPR	S183	0 ± 0	2	0 ± 0	2
LIESTSTMDGAIAAALLMFGDAGGGPRK	S183, S185	0 ± 0	2	1 ± 0	2
LKLEQDM(Oxidation (M))TTVDEGDEGSM(Oxidation (M))PADIATLLK	S1011	11 ± 7	2	0 ± 0	1
LKLEQDM(Oxidation (M))TTVDEGDEGSMPADIATLLK	S1011	2 ± 1	2	0 ± 0	1
LKLEQDMTTVDEGDEGSM(Oxidation (M))PADIATLLK	S1011	2 ± 2	2	0 ± 0	2
LKLEQDMTTVDEGDEGSMPADIATLLK	S1011	4 ± 3	2	0 ± 0	1
LQTLKELFPQRSDNDLLK	S173	0 ± 0	2	0 ± 0	1
LSSSSEPYEEDEFNDDQSIK	S211, S212, S213, S214, S227	51 ± 16	4	10 ± 5	4
LSSSSEPYEEDEFNDDQSIKK	S211, S212, S213, S214, S227	63 ± 17	5	19 ± 14	5
LSSSSEPYEEDEFNDDQSIKKTR	S212, S214	65 ± 0	1	34 ± 0	1
M(Oxidation (M))FSSKTK	S691, S692, T694	45 ± 5	2	0 ± 0	1
MFSSKTK	S691, S692, T694	14 ± 2	2	0 ± 0	1
NDDISELEDLSELEDLK	S146, S152	10 ± 3	3	2 ± 2	3
NDDISELEDLSELEDLKDAK	S146, S152	27 ± 40	5	2 ± 2	5
NKIEEAPEATPQPSQPGPSSPISLSAEEENAEGEVSR	S33, S34, S37, S39	56 ± 0	1	10 ± 0	1
NQRGIQYIDLSSDSEDVVSPNCSNTVQEK	S95, S96, S98, S103	62 ± 1	2	0 ± 0	1
QESIVLK	S254	0 ± 0	2	1 ± 1	2
QESIVLKLQK	S254	0 ± 0	2	0 ± 0	2
RKLSSSSEPYEEDEFNDDQSIK	S211, S212, S213	0 ± 0	1	64 ± 0	1
RNDDISELEDLSELEDLK	S146, S152	7 ± 6	3	0 ± 2	3
RNDDISELEDLSELEDLKDAK	S146, S152	41 ± 18	5	14 ± 10	5
RVVEDSEYDSGSDVGSSLDEDYSSGEEVM(Oxidation (M))EDGYK	S362, S368, S369, S375, S376	70 ± 7	3	13 ± 13	3
RVVEDSEYDSGSDVGSSLDEDYSSGEEVM(Oxidation (M))EDGYKGK	S362, S364, S368, S369, Y374, S375, S376	91 ± 11	3	35 ± 22	3
RVVEDSEYDSGSDVGSSLDEDYSSGEEVM(Oxidation (M))EDGYKGK	S368, S369, Y374, S375, S376	0 ± 0	1	17 ± 0	1
RVVEDSEYDSGSDVGSSLDEDYSSGEEVMEDGYK	S362, S368, S369, S375, S376	32 ± 22	3	5 ± 6	3
RVVEDSEYDSGSDVGSSLDEDYSSGEEVMEDGYKGK	S362, S364, S368, S369, Y374, S375, S376	31 ± 1	3	15 ± 14	3
RVVEDSEYDSGSDVGSSLDEDYSSGEEVMEDGYKGK	S368, S369, Y374, S375, S376	0 ± 0	1	19 ± 0	1
TEDSSVPETPDNER	S66, S67, S71	6 ± 9	2	2 ± 1	2
TEDSSVPETPDNERK	S66, S67, T71	6 ± 4	4	3 ± 3	4
TEDSSVPETPDNERKASISYFK	S66, S67, T71	37 ± 5	2	2 ± 0	1
TFNKDTVIIVSEPSEDEESQGLPTM(Oxidation (M))AR	S124, S127, S132	98 ± 1	3	19 ± 0	1
TFNKDTVIIVSEPSEDEESQGLPTMAR	S124, S127, S132	72 ± 24	4	4 ± 7	4
TFNKDTVIIVSEPSEDEESQGLPTMARR	S124, S127	91 ± 1	2	7 ± 0	1
TKSADEQSIYEK	S696	35 ± 0	1	0 ± 0	1
TRLDHGEESNESAESSSNWEK	S239, S242, S245, S246	47 ± 19	5	17 ± 13	5
TRLDHGEESNESAESSSNWEKQESIVLK	S239, S242, S245, S254	56 ± 6	3	19 ± 13	3
VFAEDQDM(Oxidation (M))QYVSQSEVPNGK	S302	10 ± 14	2	0 ± 0	2
VFAEDQDMQYVSQSEVPNGK	S302	0 ± 0	2	0 ± 0	2
VVEDSEYDSGSDVGSSLDEDYSSGEEVM(Oxidation (M))EDGYK	S358, S364, S368, S369, S376	33 ± 47	2	11 ± 9	2
VVEDSEYDSGSDVGSSLDEDYSSGEEVM(Oxidation (M))EDGYKGK	Y360, S362, S364, S376	50 ± 0	1	14 ± 0	1
VVEDSEYDSGSDVGSSLDEDYSSGEEVMEDGYK	S358, S364, S368, S369, S376	14 ± 20	2	2 ± 2	2

Table S1 Continued		(- CIP)		(+ CIP)	
**Peptide**	**Phosphorylation Sites**	**% Phosphorylation**	**n**	**% Phosphorylation**	**n**
VVEDSEYDSGSDVGSSLDEDYSSGEEVMEDGYKGK	S358, Y360, S362, S364, S368, S375, S376	36 ± 6	3	3 ± 4	2
ANTPDSDITEKTEDSSVPETPDNER	T54, T60, T63, T71	-	0	52 ± 1	2
LSSSSEPYEEDEFNDDQSIK	S213	-	0	1 ± 0	1
TFNKDTVIIVSEPSEDEESQGLPTM(Oxidation (M))ARR	S124	-	0	25 ± 0	1
LIESTSTM(Oxidation (M))DGAIAAALLMFGDAGGGPR	S183	-	0	1 ± 0	1
LIESTSTM(Oxidation (M))DGAIAAALLMFGDAGGGPRK	S185	-	0	2 ± 0	1
LIESTSTMDGAIAAALLM(Oxidation (M))FGDAGGGPRK	S185	-	0	47 ± 0	1
EVLKEHEWM(Oxidation (M))YTEALESLK	T283	-	0	0 ± 0	1
LIESTSTMDGAIAAALLM(Oxidation (M))FGDAGGGPR	S183	-	0	1 ± 0	1
LIESTSTM(Oxidation (M))DGAIAAALLM(Oxidation (M))FGDAGGGPRK	S183	-	0	67 ± 0	1
LIESTSTM(Oxidation (M))DGAIAAALLMFGDAGGGPRK	S183	-	0	2 ± 0	1
LIESTSTMDGAIAAALLM(Oxidation (M))FGDAGGGPRK	S183	-	0	54 ± 0	1
LIESTSTM(Oxidation (M))DGAIAAALLM(Oxidation (M))FGDAGGGPR	S183	-	0	2 ± 0	1
LIESTSTM(Oxidation (M))DGAIAAALLMFGDAGGGPR	S183	-	0	1 ± 0	1
LIESTSTMDGAIAAALLM(Oxidation (M))FGDAGGGPR	S183	-	0	1 ± 0	1
LIESTSTMDGAIAAALLMFGDAGGGPR	S183	-	0	1 ± 0	1
QVTM(Oxidation (M))LTGNGGGWNIEQPSILNQSLSLKPYQK	T474	-	0	19 ± 0	1
QVTMLTGNGGGWNIEQPSILNQSLSLKPYQK	T474	-	0	11 ± 0	1

Table S1. The percent phosphorylation of all WT SMARCAD1 peptides

Table of all phosphorylated (-CIP) and dephosphorylated (+CIP) WT SMARCAD1 peptides graphed in Figure S13.
